# Supplementary material for: Perinatal asphyxia and associated factors among neonates admitted to a specialized public hospital in South Central Ethiopia: A retrospective cross-sectional study
Source: PLoS One. 2022 Jan 13;17(1):e0262619. doi: 10.1371/journal.pone.0262619 (PMC8758104; doi:10.1371/journal.pone.0262619)
Supplement: Attachment — Submitted filename: PONE-D-21-06937_.minor corrections.pdf [file pone.0262619.s007.pdf]

PLOS ONE

Perinatal asphyxia and associated factors among neonates admitted to a specialized public hospital in South Central Ethiopia: A retrospective cross-sectional study

--Manuscript Draft--

**Manuscript Number:**	PONE-D-21-06937R2
**Article Type:**	Research Article
**Full Title:**	Perinatal asphyxia and associated factors among neonates admitted to a specialized public hospital in South Central Ethiopia: A retrospective cross-sectional study
**Short Title:**	Perinatal asphyxia and associated factors among neonates admitted to a specialized public hospital
**Corresponding Author:**	Abel Tibebu Goshu, MSc<br>Haramaya University College of Health and Medical Sciences<br>Harar, ETHIOPIA
**Keywords:**	Neonate; Perinatal asphyxia; NICU; Worabe; Ethiopia
**Abstract:**	Introduction: Perinatal asphyxia continues to be a significant clinical concern around the world as the consequences can be devastating. World Health Organization data indicates perinatal asphyxia is encountered amongst 6 - 10 newborns per 1000 live full-term birth, and the figures are higher for low and middle-income countries. Nevertheless, studies on the prevalence of asphyxia and the extent of the problem in poorly resourced southern Ethiopian regions are limited. This study aimed to determine the magnitude of perinatal asphyxia and its associated factors. Methods: A retrospective cross-sectional study design was used from March to April 2020. Data was collected from charts of neonates who were admitted to NICU from January 2016 to December 31, 2019. Result: The review of 311 neonates' medical records revealed that 41.2% of the neonates experienced perinatal asphyxia. Preeclampsia during pregnancy (AOR= 6.2, 95%CI :3.1- 12.3), antepartum hemorrhage (AOR= 4.5, 95%CI:2.3- 8.6), gestational diabetes mellitus (AOR= 4.2, 95%CI:1.9-9.2), premature rupture of membrane (AOR= 2.5, 95%CI:1.33-4.7) fetal distress (AOR= 3,95%CI:1.3-7.0) and meconium-stained amniotic fluid (AOR= 7.7, 95%CI : 3.1-19.3) were the associated factors. Conclusion: Substantial percentages of neonates encounter perinatal asphyxia, causing significant morbidity and mortality. Focus on early identification and timely treatment of perinatal asphyxia in hospitals should, therefore, be given priority.
**Order of Authors:**	Seifu Awgchew Mamo
	Girum Sebsibie Teshome
	Tewodros Tesfaye
	Abel Tibebu Goshu, MSc
**Opposed Reviewers:**	
**Response to Reviewers:**	Responses for the Academic editor<br>Comment 1: For the reader an explanation of the 3 delays model would be helpful.<br>Response 1: We respectfully accepted the comment. It was briefly explained in the Discussion.<br>Comment 2: Please give more detailed information regarding possible different infections of the fetus and the neonate.<br>Response 2: We respectfully accepted the comment. We have noted that the statement put in the Discussion was ambigious. However the statemet was put as a rationale for the similarity between ours and other studies. We also have tried to correct it as such on the revised document.<br>Comment 3: What umbilical cord compromises have occured in this patient sample?<br>Response 3: We thank you for the question. We have noted that the statement put in the Discussion was ambigious. However the statemet was placed as a rationale for the similarity between ours and other studies. We also have tried to correct it as such on the revised document.<br>Comment 4: What percent of reduction of neonatal mortality do you aim for, what is

Powered by Editorial Manager® and ProduXion Manager® from Aries Systems Corporation

feasible?
Response 4: We thank you for the question. In alignment with the sustainable development goals, we aim for a 35-40% reduction in the magnitude of perinatal asphyxia at WCHS by the end of the decade.
Comment 5: Please explain the capabilities of the different nurseries, especially the NICU - levels of care 1-4.
Response 5: We respectfully accepted the comment. It was briefly discussed in the Methods section, 'Study area' sub-section of the revised document.
Comment 6: Most importantly: Please review your paper, most of all the discussion, for unnecessary redundancies, length of text.
Response 6: We respectfully accepted the comment. We have tried to omit redundancies throughout the discussion section.
Thank you again!
Responses for Reviewer 1
Comment 1: Much improved manuscript.
Response 1: We are thankful once again for taking your time to refine our work.
Comment 2: Line 26 - delete "in the world" as this is repetitive.
Response 2: We respectfully accepted the comment. We have omitted the phrase.
Comment 3: Line 334 - change to read "Utilization of the APGAR alone to diagnoses perinatal asphyxia may not..."
Response 3: We respectfully accepted the comment. We have corrected it as per your recommendation.
Thank you again!

Responses for Reviewer 2
Comment 1: The paper has been significantly improved after revising. It covers an important topic and is well written.
Response 1: We thank you for the encouraging words and the time you have spent in refining the document.
Comment 2: In Abstract (page 2 lines 29-30), the authors stated that " ...studies on the prevalence of asphyxia in these countries are limited." The above statement is incorrect. This is not new information. Many other similar studies have been conducted in Ethiopia and published. It is unclear how this study specifically contributes to the literature. Suggest double-checking the similarity report too.
Response 2: We respectfully accepted the comment. As per your recommendation, a correction was made to the specific statement that indicated there is limited information on the topic. We also believe the study's findings will add to the body of knowledge interms of illuminating the problem in the study area. Furthermore, the findings of this study will direct responsible federal and local stakeholders to design effective implementations to reduce the magnitude of perinatal asphyxia.
Comment 3: Suggest consolidating the Introduction. The operational definition should be elaborated further in this section (not Methodology).
Response 3: We respectfully accepted the comment. We added a paragraph on the diagnostic definitions of perinatal asphyxia in the Introduction. Further elaboration was added in the methods section, as there was a comment by other reviewer.
Comment 4: Methodology-A citation is needed for the study area and period (Page 4, line 97)
Response 4: We respectfully accepted the comment. A citation is added to the description of the study area.
Comment 5: Please revise the inclusion criteria as it is similar to the source population. (page 5, line 105 & line 110). Please take note that "2016 to December 31, 2019" appeared three times in Methods.
Response 5: We respectfully accepted the comment. We have tried to reduce the

	redundancies by compiling only the necessary information under the sub section 'Study population'.<br>Comment 6: Please remove this statement "The overall prevalence of perinatal asphyxia among neonates who were delivered at WCHS was found to be 128(41.2%) from Clinical Characteristics" (page11, lines 204-205). You have reported it under the magnitude of perinatal asphyxia (Page 12, lines 228-229).<br>Response 6: We respectfully accepted the comment. As per your recommendation, the statement is removed in the revised editon.<br>Comment 7: The Discussion has improved. Suggest to re-write Limitation because it does not appear to be sound.<br>Response 7: We respectfully accepted the comment. We have tried to modify the Limitation in the revised edition.<br>Comment 8: The conclusion can be further enhanced by adding recommendations for future research.<br>Response 8: We respectfully accepted the comment. We added recommendations for future researchers in the revised document.<br>Thank you again!
**Additional Information:**	
**Question**	**Response**
**Financial Disclosure**<br><br>Enter a financial disclosure statement that describes the sources of funding for the work included in this submission. Review the submission guidelines for detailed requirements. View published research articles from *PLOS ONE* for specific examples.<br><br>This statement is required for submission and **will appear in the published article** if the submission is accepted. Please make sure it is accurate.<br><br>**Unfunded studies**<br>Enter: *The author(s) received no specific funding for this work.*<br><br>**Funded studies**<br>Enter a statement with the following details:<br>• Initials of the authors who received each award<br>• Grant numbers awarded to each author<br>• The full name of each funder<br>• URL of each funder website<br>• Did the sponsors or funders play any role in the study design, data collection and analysis, decision to publish, or preparation of the manuscript?<br>• **NO** - Include this sentence at the end of your statement: *The funders had no role in study design, data collection and analysis, decision to publish, or preparation of the manuscript.*<br>• **YES** - Specify the role(s) played.	The authors received no specific funding for this work.

* typeset	
**Competing Interests**<br><br>Use the instructions below to enter a competing interest statement for this submission. On behalf of all authors, disclose any competing interests that could be perceived to bias this work—acknowledging all financial support and any other relevant financial or non-financial competing interests.<br><br>This statement is required for submission and **will appear in the published article** if the submission is accepted. Please make sure it is accurate and that any funding sources listed in your Funding Information later in the submission form are also declared in your Financial Disclosure statement.<br><br>View published research articles from *PLOS ONE* for specific examples.<br><br>**NO authors have competing interests**<br><br>Enter: *The authors have declared that no competing interests exist.*<br><br>**Authors with competing interests**<br><br>Enter competing interest details beginning with this statement:<br><br>*I have read the journal's policy and the authors of this manuscript have the following competing interests: [insert competing interests here]*<br><br>* typeset	The authors have declared that no competing interests exist.
**Ethics Statement**<br><br>Enter an ethics statement for this submission. This statement is required if the study involved:	Ethical clearance for the commencement of the study was obtained from the School of Nursing and Midwifery Research Ethical Committee. Permission was also sought and obtained from the ethical committee of Worabe comprehensive specialized hospital. Data was kept anonymous by keeping the identity of the neonate's or mother's credentials hidden.

• Human participants<br>• Human specimens or tissue<br>• Vertebrate animals or cephalopods<br>• Vertebrate embryos or tissues<br>• Field research<br><br>Write "N/A" if the submission does not require an ethics statement.<br><br>General guidance is provided below. Consult the submission guidelines for detailed instructions. **Make sure that all information entered here is included in the Methods section of the manuscript.**	

**Format for specific study types**<br><br>**Human Subject Research (involving human participants and/or tissue)**<br>• Give the name of the institutional review board or ethics committee that approved the study<br>• Include the approval number and/or a statement indicating approval of this research<br>• Indicate the form of consent obtained (written/oral) or the reason that consent was not obtained (e.g. the data were analyzed anonymously)<br><br>**Animal Research (involving vertebrate animals, embryos or tissues)**<br>• Provide the name of the Institutional Animal Care and Use Committee (IACUC) or other relevant ethics board that reviewed the study protocol, and indicate whether they approved this research or granted a formal waiver of ethical approval<br>• Include an approval number if one was obtained<br>• If the study involved *non-human primates*, add *additional details* about animal welfare and steps taken to ameliorate suffering<br>• If anesthesia, euthanasia, or any kind of animal sacrifice is part of the study, include briefly which substances and/or methods were applied<br><br>**Field Research**<br><br>Include the following details if this study involves the collection of plant, animal, or other materials from a natural setting:<br>• Field permit number<br>• Name of the institution or relevant body that granted permission	
**Data Availability**<br><br>Authors are required to make all data underlying the findings described fully available, without restriction, and from the time of publication. PLOS allows rare exceptions to address legal and ethical concerns. See the PLOS Data Policy and FAQ for detailed information.	Yes - all data are fully available without restriction

A Data Availability Statement describing where the data can be found is required at submission. Your answers to this question constitute the Data Availability Statement and **will be published in the article**, if accepted.<br><br>**Important:** Stating 'data available on request from the author' is not sufficient. If your data are only available upon request, select 'No' for the first question and explain your exceptional situation in the text box.<br><br>Do the authors confirm that all data underlying the findings described in their manuscript are fully available without restriction?	
**Describe where the data may be found in full sentences. If you are copying our sample text, replace any instances of XXX with the appropriate details.**<br><br>• If the data are **held or will be held in a public repository**, include URLs, accession numbers or DOIs. If this information will only be available after acceptance, indicate this by ticking the box below. For example: *All XXX files are available from the XXX database (accession number(s) XXX, XXX.).*<br>• If the data are all contained **within the manuscript and/or Supporting Information files**, enter the following: *All relevant data are within the manuscript and its Supporting Information files.*<br>• If neither of these applies but you are able to provide **details of access elsewhere**, with or without limitations, please do so. For example:<br><br>*Data cannot be shared publicly because of [XXX]. Data are available from the XXX Institutional Data Access / Ethics Committee (contact via XXX) for researchers who meet the criteria for access to confidential data.*<br><br>*The data underlying the results presented in the study are available from (include the name of the third party*	All relevant data are within the manuscript.

and contact information or URL).<br>• This text is appropriate if the data are owned by a third party and authors do not have permission to share the data.<br><br>* typeset	
Additional data availability information:	

November 5, 2021

Dear Editorial team,

We are submitting the second revised draft of an original research article entitled "Perinatal asphyxia and associated factors among neonates admitted to a specialized public hospital in South Central Ethiopia: A retrospective cross-sectional study" for consideration by the PLOS ONE journal.

The authors confirm that this work is original and has not been published elsewhere, nor is it currently under consideration for publication elsewhere.

This paper details the magnitude of perinatal asphyxia and its determining factors in one of the poorly resourced areas of Ethiopia. It also magnifies the challenges faced and alerts responsible stakeholders about the essence of early recognition and correct assessment and management of infants with prenatal asphyxia.

We believe that this manuscript is appropriate for publication because our objectives align with PLOS ONE's aims and scopes.

We have no conflicts of interest to disclose. Please address all correspondence concerning this manuscript to:-

Abel Tibebu Goshu
Email: ABTI2222@GMAIL.COM
Phone: +251913243086

Thanks for the consideration.

Sincerely,

The Authors

Manuscript Click here to access/download;Manuscript;Manuscript.docx

Perinatal asphyxia and associated factors among neonates admitted to a specialized public hospital in South Central Ethiopia: A retrospective cross-sectional study

Seifu Awgchew Mamo [1¶], Girum Sebsibie Teshome [2¶], Tewodros Tesfaye[2¶], Abel Tibebu Goshu[3¶*]

[1]Department of Pediatric Nursing, College of Medicine and Health sciences, Wachemo University, Hosaena, Ethiopia

[2]School of Nursing and Midwifery, College of Health Sciences, Addis Ababa University, Addis Ababa, Ethiopia

[3]School of Nursing and Midwifery, College of Health and Medical sciences, Haramaya University, Harar, Ethiopia

*Corresponding Author

Abel Tibebu Goshu

Email: ABTI2222@GMAIL.COM

¶ - These authors contributed to this work equally.

Abstract

Introduction: Perinatal asphyxia continues to be a significant clinical concern around the world as the consequences can be devastating. World Health Organization data indicates perinatal asphyxia is encountered amongst 6 - 10 newborns per 1000 live full-term birth, and the figures are higher for low and middle-income countries. Nevertheless, studies on the prevalence of asphyxia and the extent of the problem in poorly resourced southern Ethiopian regions are limited. This study aimed to determine the magnitude of perinatal asphyxia and its associated factors. **Methods:** A retrospective cross-sectional study design was used from March to April 2020. Data was collected from charts of neonates who were admitted to NICU from January 2016 to December 31, 2019. **Result:** The review of 311 neonates' medical records revealed that 41.2% of the neonates experienced perinatal asphyxia. Preeclampsia during pregnancy (AOR= 6.2, 95%CI :3.1- 12.3), antepartum hemorrhage (AOR= 4.5, 95%CI:2.3- 8.6), gestational diabetes mellitus (AOR= 4.2, 95%CI:1.9-9.2), premature rupture of membrane (AOR= 2.5, 95%CI:1.33-4.7) fetal distress (AOR= 3,95%CI:1.3-7.0) and meconium-stained amniotic fluid (AOR= 7.7, 95%CI : 3.1-19.3) were the associated factors. **Conclusion:** Substantial percentages of neonates encounter perinatal asphyxia, causing significant morbidity and mortality. Focus on early identification and timely treatment of perinatal asphyxia in hospitals should, therefore, be given priority.

Keywords: Neonate, Perinatal asphyxia, NICU, Worabe, Ethiopia

Introduction

A decade remains to achieve the third Sustainable Development Goal (SDG) targets, including reducing preventable causes of neonatal mortalities (1, 2). Though the number of neonatal deaths has declined significantly within the last thirty years, Africa alone accounted for about 41% of the total global neonatal mortality in the year 2017 (3).

Perinatal asphyxia, also called birth asphyxia, is one of the leading causes of neonatal deaths in the world following severe infections and prematurity (1, 4). It results from the loss of the blood supply or impairment of gas exchange to or from the fetus before, during, or after the birth process (5). Perinatal asphyxia may lead to severe metabolic acidosis, hypercarbia, progressive hypoxemia, neonatal encephalopathy, and multi-system organ failure, and even result in death (6-10).

An arterial blood sample, APGAR score (appearance, pulse, grimaces, activity, and respiration), immediate neurological complications, or evidence of multiple organ dysfunction are used to diagnose asphyxia. The APGAR score is assessed in the first and fifth minutes of life and ranges from zero to ten. According to the WHO classification, an APGAR score of four to seven in the first minute of life indicates moderate prenatal asphyxia, whereas zero to three suggests severe asphyxia(11, 12). Factors related to antepartum, intrapartum or immediate postpartum period may contribute to perinatal asphyxia development (13). These could be summarized into abnormal maternal oxygenation, congenital infections, insufficient placental perfusion, traumatic deliveries, or impaired umbilical circulation. Most studies identified that intrapartum factors as having the highest impact (7, 14, 15).

Worldwide, perinatal asphyxia is encountered amongst 6 - 10 newborns per 1000 live full-term birth (9, 16, 17). Apparently, the numbers are higher for low and middle-income countries. In Africa, significant neonatal morbidity and mortality occur due to complications associated with perinatal asphyxia. Studies from different regions of the continent show variant figures on the magnitude. A study from two district hospitals in Ghana showed that birth asphyxia to be the second cause of admission (15.1%) and the third cause of mortality (20.7%). In contrast, another study conducted in Nigeria indicated severe perinatal asphyxia to be the most important cause of death in all birth weight categories except in extremely low

birth weight babies (18, 19). A systematic review conducted amongst east and central African countries has shown a pooled prevalence of perinatal asphyxia to be 15.9% (9).

Ethiopian studies are no different from the rest of Africa. Though the country has made efforts to minimize under five and neonatal mortality rates by devising a National strategy for Newborn and Child Survival, current figures still show demand for committed action to target and tackle preventable causes like birth asphyxia (20, 21). The Ethiopian Demographic and Health survey in 2019 revealed a reduction in early childhood mortality except for neonatal mortality, which had steady progress since 2016. It was shown that birth asphyxia was and still is the primary cause of neonatal deaths (22). Observational studies in different parts of the country also magnify this concern. A cross-sectional study conducted in the Tigray region of northern Ethiopia showed that 22.1% of the total neonates included in the study experienced perinatal asphyxia (23). At the same time, another study from Dilla referral hospital revealed the prevalence of perinatal asphyxia to be 32.8% (24).

In order to improve the quality of care that is delivered within a country, one must first understand the problem and the issues that healthcare providers encounter. Improving the care of the maternal-infant dyad can lead to a significant decrease in the rates of perinatal asphyxia (22, 25). In an effort to enhance care in Ethiopia, we sought to understand the burden of perinatal asphyxia and early assessment practices. To do this, we evaluated cases of perinatal asphyxia and identified associated factors among neonates admitted to the neonatal intensive care unit of Worabe Comprehensive Specialized Hospital (WCSH) in southern Ethiopia.

Methods

Study area and period

The study was conducted at Worabe Comprehensive Specialized Hospital (WCSH), Silte zone, south central Ethiopia, from March to April 2020 (by reviewing neonates' charts from January/2016 to December 31/2019). WCSH was established in October 2014 and is located 170 km southwest of Addis Ababa. It is the only referral hospital in Silte zone and provides emergency, out-patient and in-patient services to the

local and neighboring communities. The Neonatal Intensive Care Unit has 18 beds, 5 incubators, 15 radiant warmers and operates with 17 nurses, 2 midwives, 1 general practitioner, 2 pediatricians and 1 gynecologist. The NICU offers diagnostic and treatment services for approximately 1000-1500 newborns per year (26). The level of newborn care is determined by the neonate's gestational age, birth weight, sickness severity, and the facility's general setup. Accordingly, health practitioners at the level I offer basic newborn care to low-risk infants and triage unwell newborns. Specialty treatment (Level II NICU) is confined to newborn infants over 32 weeks gestational age weighing 1500 g or recovering from severe disease treated in a level III environment (subspecialty). All newborn infants with extreme preterm (28 weeks or less) or extremely low birth weight (1000g or less), or severe and/or complex disease are handled in level III NICU (26).

Study design

A facility-based retrospective cross-sectional study design was employed.

Population

Source population

All records' of neonates who were admitted at NICU of Worabe comprehensive specialized hospital.

Study population

All the records' of neonates who were admitted to the NICU of WCSH were added to the study. The records' of neonates with incomplete documentation (which has no proper maternal or fetal measurement parameters), with major congenital malformations or anomalies were excluded.

Sample size determination

The sample size was calculated using the single population proportion formula considering the p-value of 32.8% from a previous study conducted in Ethiopia (24)

$$ni = \frac{(z\alpha/2)^2 * p\,(1 - p)}{d^2}$$

Where, ni = initial sample size, p = proportion of prenatal asphyxia; 32.8% = 0.328, $(z\alpha/2)^2$= confidence interval (95%), d= is the margin of sampling error tolerated (5%) = 0.05

Substituting the values for each of these variables in the above formula, the sample size was estimated to be 339.

Because the source population is less than ten thousand, we used a correction formula

$$nf = ni/ (1+ni/N)$$

Where nf= final sample size

ni = calculated sample size (initial sample size)

N= total number of neonates admitted to NICU of WCSH within the three years period (3796 neonates)

$$nf = 339/(1+339/3796) = 311$$

Sampling procedure

Systematic random sampling technique was used to select which charts to review. After the card records of the neonates were put in their order of admission, the kth interval was determined by dividing the total population size by the total sample size.

$$k = N/nf=3796/311=12$$

Where, N is the total population at NICU of WCSH from January /2016 to December 31/2019,

nf = final sample size of the study).

Operational definitions

Perinatal asphyxia – is the inability of a newborn to initiate and sustain respiration by persistently scoring an APGAR score of less than 7 for more than 5 minutes after delivery (24, 27).

Prolonged labor – is the first stage of labor, exceeding 12 hours in primigravida or 8 hours in multipara mothers (25).

Premature rupture of membranes (PROM) – rupture of the amniotic sac and chorion membrane occurred before the onset of labor (24).

Study variables

Magnitude of perinatal asphyxia was the outcome variable. Maternal socio-demographic variable (age in year, educational status, religion, residency, and occupation), antepartum related variable (anemia during Pregnancy, ANC follow up, DM, pregnancy-induced hypertension, antepartum hemorrhage, chronic hypertension, gravidity, and parity), intrapartum related variable (PROM, prolonged labor, obstructed labor, place of delivery and mode of delivery), neonatal related variable (birth weight, fetal distress, the gender of the neonate and intrauterine meconium release) were independent variables.

Data collection tools and procedures

Data was collected from neonates' registration and medical records using a structured checklist adapted and modified from different literature(13, 23, 24, 28). The checklist contained four sections which assess socio-demographic, antepartum factors (parity/gravidity, gestational age, complications/illnesses during pregnancy), intrapartum factors (mode of delivery, duration of labor, fetal presentation, fetal distress, intrauterine meconium release occurrence of PROM, and obstructed labor) and neonatal factors (sex, birth weight, gestational age at birth and occurrence of perinatal asphyxia). To identify maternal-related factors, the maternal record chart of the same year was traced. Three BSc nurses were recruited as data collectors and were trained for two days on the checklist, data collection, and supervision. Furthermore, the sampling procedures were also elucidated to them. The checklist was pretested for its relevance and clarity to answer the research question.

Ethical Considerations

Ethical clearance for the commencement of the study was obtained from the Addis Ababa University, School of Nursing and Midwifery Research Ethical Committee (Ref. no. 030/20/SNM). Permission was also sought and obtained from the ethical committee of Worabe comprehensive specialized hospital. Data was kept anonymous by keeping the identity of the neonate's or mother's credentials hidden before, during, and after the study.

Data analysis and management

The data were entered into statistical software Epi-data version 3.1. The entered data was subject to cleaning using simple frequency and tabulation to ensure the data's validity. Then the analysis was made with IBM SPSS version 24.0 after exporting the prepared data. Descriptive statistics, such as frequency distribution, were computed to describe the significant variables of the study. Odds ratio and the p-value were computed. $P \leq 0.05$ was considered as statistically significant for association. Binary logistic regression was conducted to see the effect of each of the independent variables on the outcome variable, and variables that were statistically significant at P value less than 0.05 levels were put into the final model (multivariate analysis) to control for confounding.

Results

Socio-demographic characteristics of the mothers' of neonates

Data were collected from a total of 311 neonatal medical records. Of the neonates' mothers, 105(33.8%) were between the ages of 25-29 years, and 163(52.4%) of the mothers had no formal education. Almost all the mothers, 297 (95.5%), were Muslim by religion **(Table 1).**

Table.1 Socio-demographic characteristic of mothers of newborns at WCSH, South central Ethiopia (n=311)

Variable	Category	Frequency/ Percentage of mothers’ of neonates (n=311)	Frequency/Percentage of mothers’ of asphyxiated neonates (n=128)
Age	≤19yr	12 (3.9%)	6 (4.68%)
	20-24yr	65 (20.9)	24 (18.75%)
	25-29yr	105 (33.8%)	44 (34.37%)
	30-34yr	84 (27%)	34 (26.56%)
	≥35	45 (14.5%)	20 (15.625%)
Educational status	No formal education	163 (52.4%)	78 (60.93%)
	Primary	64 (20.6%)	24 (18.75%)
	Secondary	45 (14.5%)	14 (10.93%)
	Higher education	39 (12.5%)	12 (9.37%)
Residence	Rural	207 (66.6%)	93 (72.65%)
	Urban	104 (33.4%)	35 (27.34%)
Religion	Muslim	297 (95.5%)	123 (96%)
	Orthodox	10 (3.2%)	3 (2.34%)
	Protestant	4 (1.3%)	2 (111.56%)
	Catholic	0 (0%)	0 (0%)
Occupation status	Housewife	120 (38.6%)	57 (44.53%)
	Private	2 (0.6%)	1 (0.78%)
	Government	63 (20.3%)	23 (17.96%)
	merchant	112 (36%)	46 (35.93%)
	Student	14 (4.5%)	1 (0.78%)

Obstetrics history of the mothers at WCSH

Regarding parity, 196(63%) mothers were multiparous, and 197(63.3%) of them were attending their antenatal follow up. Among the mothers, 129(41.5%) had anemia, while 86(27.7%) were diagnosed with preeclampsia. Only 61(19.6%) of mothers had gestational diabetes mellitus, and few mothers, 21(6.8%), had chronic hypertension. Of the 311 mothers, 92(29.6%) had an antepartum hemorrhage, and 222(71.4%) mothers of the neonates delivered by SVD. Concerning membrane status, 100(32.2%) mothers developed premature rupture of the membrane, and 115(37%) mothers had obstructed labor. **(Table 2)**

Table-2 Obstetrics history of mothers at WCSH, South central Ethiopia (n=311)

Variable	Category	Frequency(n=311)	Percentage (%)
Parity	Primipara	115	37%
	Multipara	196	63%
Gestational age	<37weeks	43	13.8%
	>42weeks	18	5.8%
	37week-42week	250	80.4%
ANC[a] follow up	Yes	197	63.3%
	No	114	36.7%
Anemia	Yes	129	41.5%
	No	182	58.5%
Preeclampsia/eclampsia	Yes	86	27.7%
	No	225	72.3%
Chronic hypertension	Yes	21	6.8%
	No	290	93.2%
APH[b]	Yes	92	29.6%
	No	219	70.4%
DM	Yes	24	7.7%
	No	287	92.3%
Gestational DM[c]	Yes	61	19.6%
	No	250	80.4%
Chronic illness	Yes	247	79.4%
	No	64	20.6%
Mode of delivery	SVD[d]	222	71.4%
	C/S[e]	72	23.2%
	Instrumental	17	5.5%
Duration of labor	>12hr(prolong)	95	30.5%
	<12hr	216	69.5%
PROM[f]	Yes	100	32.2%
	No	211	67.8%
Obstructed labor	Yes	115	37%
	No	196	63%

[a]Antenatal Care, [b]Antepartum Hemorrhage, [c]Diabetes mellitus, [d]Spontaneous Vaginal Delivery, [e]Cesearian Section, [f]Prematue Rupture of Membrane

Clinical characteristics of neonates at WCSH

From the total number of neonates, 192(61.7%) of them were males, and 119 (38.3%) were females, and most of the neonates, 240(77.2%), had birth weight between 2.5kg to 4kg. Only 84(27%) of babies passed their meconium during labor, and 119(38.3%) of them developed fetal distress. **(Table 3)**

Table-3 Clinical characteristics of neonates at WCSH, South central Ethiopia (n=311)

**Characteristics**	**Category**	**Frequency(n=311)**	**Percentage (%)**
Presentation	Vertex	251	80.7%
	Non vertex	60	19.3%
Place of delivery	Home	10	3.2%
	Health center	157	50.5%
	Private clinic	3	1%
	Hospital	141	45.5 %
Gender	Male	192	61.7%
	Female	119	38.3%
Birth weight	<2.5kg	59	19%
	2.5kg-4kg	240	77.2%
	>4kg	12	3.9%
Intrauterine meconium release	Yes	84	27%
	No	227	73%
Fetal distress	Yes	119	38.3%
	No	192	61.7%
Perinatal asphyxia	Yes	128	41.2%
	No	183	58.8%

Magnitude of perinatal asphyxia

The overall prevalence of perinatal asphyxia among neonates who were delivered at WCHS was found to be 128(41.2%).

Factors associated with the occurrence of perinatal asphyxia

Different socio-demographic, neonatal, and maternal variables were tested for their association with the presence of perinatal asphyxia. For the multivariate logistic regression, all variables that were found to have an association with the outcome variable in bivariate logistic regression at P=0.25 were included in the multivariate logistic regression models. During multivariate logistic regression, six variables (preeclampsia, antepartum hemorrhage, gestational diabetes mellitus, premature rupture of membrane, intrauterine meconium release, and fetal distress during labor) were found significantly associated with perinatal asphyxia (at $p \leq 0.05$).

Among maternal factors, preeclampsia was independently associated with significantly higher odds of the newborn developing perinatal asphyxia (AOR= 6.2, 95% CI, 3.1, 12.3). Neonates from mothers who had antepartum hemorrhage were 4.5 times more affected by asphyxia as compared to neonates who delivered from mothers who had no antepartum hemorrhage(AOR= 4.5, 95% CI, 2.3, 8.6). Furthermore, neonates from mothers with gestational diabetes mellitus were four times more likely to be asphyxiated than neonates from mothers without gestational diabetes mellitus (AOR= 4.2, 95% CI 1.9,9.2). Moreover, neonates of mothers who had a history of premature rupture of the membrane were 2.5 times more likely to be asphyxiated than those who had not encountered premature rupture (AOR= 2.5, 95%CI,1.33,4.7). Among the fetal factors, fetal distress during labor and meconium-stained amniotic fluid were significantly associated with asphyxia (AOR= 3, 95%CI, 1.3.7.0) and (AOR= 7.7, 95%CI, 3.1, 19.3) respectively. **(Table 4)**

Table-4 Factors associated with perinatal asphyxia among neonates admitted at WCSH, South central Ethiopia (n=311)

Variables	Asphyxiated		COR 95%CI	AOR 95%CI	p-value
	No	Yes			
**Educational status**					
No formal education	85	78	2.07(.98, 4.4)	1.6(.5, 5.1)	0.420
Primary	40	24	1.4(.6, 3.2)	1.2(.4, 3.7)	0.624
Secondary	31	14	1.0(.4, 2.6)	1.2(.4, 3.5)	0.607
Higher education	27	12	1	1	
**Residence**					
Rural	114	93	1.6(.985,2.63)	1.0(0.4,2.3)	0.911
Urban	69	35	1	1	
**Preeclampsia/Eclampisa**					
Yes	**25**	**61**	**5.75(3.33, 9.93)**	**6.2(3.1,12.3)**	**0.000***
No	158	67	1	1	
**Antepartum hemorrhage**					
Yes	**31**	**61**	**4.464 (2.656, 7.5)**	**4.5(2.3,8.6)**	**0.000***
No	152	67	1	1	
**Gestational diabetes mellitus**					
Yes	**17**	**44**	**5.115(2.757,9.5)**	**4.2(1.9,9.2)**	**0.000***
No	166	84	1	1	
**Mode of delivery**					
SVD[a]	139	83	1	1	
CS[b]	39	33	1.417(.828,2.425)	1.0(0.5,2)	0.912
Instrumental	5	12	4.019(1.368,11.81)	2.3(0.5,9.5)	0.258
**Duration of labor**					
>12hr	49	46	1.534(.942,2.5)	1.1(0.6,2.2)	0 .703
<12hr	134	82	1	1	
**Presentation of the fetus**					
Vertex	153	98	1	1	
Non vertex	30	30	1.561(.886,2.75)	1.5(0.7,3.5)	0.311
**Premature rupture of membrane**					
Yes	**42**	**58**	**2.782(1.705,4.54)**	**2.5(1.33,4.7)**	**0.004***
No	141	70	1	1	

**Obstructed labor**					
Yes	52	63	2.442(1.522,3.92)	1.9(1.0,3.8)	0.053
No	131	65	1	1	
**Fetal distress during labor**					
Yes	**59**	**60**	**1.854(1.164,2.95)**	**3.0(1.3, 7.0)**	**0.010***
No	124	68	1	1	
**Intrauterine meconium release**					
Yes	**23**	**61**	**6.33(3.62,11)**	**7.7(3.1,19.3)**	**0.000***
No	160	67	1	1	
**Birth weight**					
< 2.5kg	40	19	1	1	
2.5kg-4kg	137	103	2.105(0.6, 7.39)	2.67(0.6, 12.6)	0.207
>4kg	6	6	1.33(.417, 4.24)	2.904(0.7,12.2)	0.146

[a]Spontaneous Vaginal Delivery, [b]Cesearian Section, *AOR= statistically significant at $p \le 0.05$

Discussion

The findings from this study revealed that the magnitude of perinatal asphyxia was 41.2%, which is quite higher compared to a study done in southern India (2.7%)(29), Sweden (5.4%) (30), Alberta Canada (2.28%)(31) and South-East Nigeria (12.8%) (32). This discrepancy could be explained by the socioeconomic variation between the study area and the other countries. Better developed countries have the necessary infrastructure and skilled health care providers to significantly reduce the incidence of perinatal complications. Nonetheless, the finding is lower than a study done in Bangladesh (56.9%) (33) and Ghana 283(61.8%) (34). The possible explanation for this variation stems from the latter two studies being conducted in poorly resourced areas. Additionally, the higher magnitude in these studies may be due to the reported low number of healthcare professionals trained to conduct neonatal resuscitation. Comparably, this study's result was higher than other studies conducted elsewhere in Ethiopia (12, 23, 28, 35, 36). The difference could be attributed to the relative urban nature of the cities, which in turn affects the quality of setup amongst the facilities, the living standards of resident mothers and accessibility of health

institutions. This is a critical indicator that the case of perinatal asphyxia needs more emphasis in the study area.

The Ethiopian ministry of health adopted the WHO recommendation of at least four ANC follow-up visits throughout a pregnancy. Despite this, ANC is utilized well in urbanized cities like Addis Ababa and Dire Dawa while semi urban and rural areas are far behind (22). This is attributed to maternal illiteracy and low socio-economic status in the latter as these factors have direct relations to the delay in health seeking behavior(25). The non-adherence to follow up could be catastrophic as it could endanger the maternal and fetal outcomes. This study revealed that 36.7% of mothers of neonates with asphyxia were not strictly attending their antenatal follow-up, paving the way for the 'silent' progress of pregnancy-induced complications (PICs) and associated perinatal outcomes.

The unpredictable nature of PICs, poor obstetric care and low service utilizations in Ethiopia, largely give rise to the maternal and perinatal sequelae. Even though the country adopted the "three delays" model, a set of strategies targeting delays in the decision to seek care, delays in seeking care, and delays in receiving adequate health care, successful implementation yet needs significant work (25). This study has identified major PICs including preeclampsia, antepartum hemorrhage, gestational diabetes mellitus, premature rupture of the membrane continue to be significant predictors of perinatal asphyxia.

Preeclampsia and eclampsia are known predictors of maternal and perinatal morbidities and mortalities (14). These disorders, categorized under Pregnancy Induced Hypertensive Disorders (PIHD), are common concerns globally, even more in low-income countries like Ethiopia. Studies have shown that variations in the prevalence of PIHDs can be observed within a country. For instance, the SNNPR region where the study area is located is one of the poorly resourced areas of the country and has a higher percentage of PIHD than any other region in the country (14). Despite the absence of novel approaches to exactly predict the occurrence of preeclampsia, complications like birth asphyxia could be lowered through frequent and consistent follow-up during pregnancy. This study reaffirmed that preeclampsia is significantly associated with perinatal asphyxia. Studies conducted in India (29), Bangkok Rajavithi hospital (37), Gusau Nigeria (38), and Tigray Ethiopia (23) are in line with this data.

Significant evidence reveals (15, 39, 40) that the occurrence of antepartum bleeding results in a decreased blood flow from the mother to the placenta with subsequent hypoxia to the fetus. Studies conducted in Ethiopia have also shown an increased incidence of perinatal asphyxia among mothers who encountered APH (15, 40). This study also identified that antepartum hemorrhage was associated with perinatal asphyxia, consistent with the study conducted in Bangalore, Indonesia (41), and Accra Ghana (34).

Gestational diabetes mellitus was also a factor associated with perinatal asphyxia, and this result is in agreement with what was reported in Canada (31), Pakistan (13), and the study done in Sweden (30). The possible explanations for these are the metabolic derangement responsible for the inadequate production of surfactants. In Ethiopia, health care providers screen and initiate counseling for lifestyle modification during the early stages of the diagnosis, followed by a prescribed insulin regimen. Nevertheless, effective implementation of these strategies once again is hindered (42, 43). The current study found out that premature rupture of the membrane was significantly associated with perinatal asphyxia which is consistent with other findings from Pakistan (30, 44), Ghana (34), and Tanzania (45). The similarity between the studies may be explained by the scientific evidence that links PROM to the incidence of oligohydramnios, resulting in possible infection and umbilical cord compression (46).

The present study identified fetal distress and meconium-stained amniotic fluid as significant factors impacting the occurrence of perinatal asphyxia. Comparable results were found from other parts of the world; Malawi (47), Thailand (37), Pakistan(44), and India(48). Studies conducted in central, south, and northern parts of Ethiopia are also in line (24, 36, 49). The rationale for the poor perinatal outcome in Ethiopia likely stems from sub-standard partograph utilization, poor intrapartum care, and ineffective neonatal resuscitation practices (50-54).

Limitations

Even though the study generated pertinent data that can be used as a baseline, the results can only describe the context in WCSH. Furthermore, the study also did not include other health lower health care facilities where deliveries were conducted. The hospital where the study was employed is an institution where

pregnant women with complications are referred to, which may have inflated the magnitude of the problem. This study utilized only the APGAR score to diagnose perinatal asphyxia. Additional investigations, such as the arterial blood gas analysis for pH, were not considered. The nature of cross-sectional study design also has an impact in defining cause and effect relationships.

Conclusion

The study revealed that a substantial percentage of neonates experienced perinatal asphyxia. Major PICs such as preeclampsia, APH, and gestational DM, PROM, and fetal factors like fetal distress and intrauterine meconium release were significantly associated with the occurrence of perinatal asphyxia. Focus on early identification and timely treatment of perinatal asphyxia in hospitals should, therefore, be given priority. Effective implementation of community engaging approaches such as mobilization and sensitization would improve the utilization of ANC services among mothers. Furthermore, revitalizing maternal waiting homes in the area may mitigate the delays in seeking care and associated complications. The Ministry of health and the hospital administrators need to devise strategies that strengthen the health care system, meliorate the obstetric care setup and ensure the adherence of health care providers to intrapartum and immediate postpartum protocols such as partograph utilization and newborn resuscitation. Researchers should focus on conducting implementation research to assess the relative impact of strengthening and revitalizing best practices.

Abbreviations: (SDG) Sustainable Development Goal, WHO (World Health Organization), WCSH (Worabe Comprehensive Specialized Hospital), SVD (Spontaneous Vaginal Delivery), ANC (Antenatal Care) NICU (Neonatal Intensive Care Unit), PROM (Premature Rupture of Membrane), DM (Diabetes Mellitus), CS (Cesarean Section), PICs (Pregnancy Induced Complications), PIHDs (Pregnancy Induced Hypertensive Disorders).

Acknowledgment

We are indebted to Worabe comprehensive specialized hospital for providing us permission to conduct the study.We would like to thank Addis Ababa University, College of Health Sciences, for the offered technical support. Finally, the authors are also thankful to the supervisors and data collectors.

Authors' contributions

All authors collaboratively made substantial contributions to conception and design, acquisition of data, or analysis and interpretation of data; took part in drafting the article or revising it critically for important intellectual content; gave final approval of the version to be published; and agree to be accountable for all aspects of the work.

Disclosure

The authors have no conflict of interest to disclose.

References

1. WHO. MCEE-WHO methods and data sources for child causes of death 2000-2016. Geneva, Switzerland2018.

2. WHO. Towards a global action plan for healthy lives and well-being for all World Health Organization; 2018. p. 37.

3. Hug L, Alexander M, You D, Alkema L. National, regional, and global levels and trends in neonatal mortality between 1990 and 2017, with scenario-based projections to 2030: a systematic analysis. The Lancet Global Health. 2019;7(6):e710-e720.

4. Oza S, Lawn JE, Hogan DR, Mathers C, Cousens SN. Neonatal cause-of-death estimates for the early and late neonatal periods for 194 countries: 2000-2013. Bull World Health Organ. 2015;93(1):19-28.

5. Goldenberg RL, Harrison MS, McClure EM. Stillbirths: The Hidden Birth Asphyxia - US and Global Perspectives. Clin Perinatol. 2016;43(3):439-453.

6. Allanson ER, Muller M, Pattinson RC. Causes of perinatal mortality and associated maternal complications in a South African province: challenges in predicting poor outcomes. BMC pregnancy and childbirth. 2015;15:37.

7. Simiyu IN, McHaile DN, Katsongeri K, Philemon RN, Msuya SE. Prevalence, severity and early outcomes of hypoxic ischemic encephalopathy among newborns at a tertiary hospital, in northern Tanzania. BMC pediatrics. 2017;17(1):131.

8. Usman F, Imam A, Farouk ZL, Dayyabu AL. Newborn Mortality in Sub-Saharan Africa: Why is Perinatal Asphyxia Still a Major Cause? Annals of global health. 2019;85(1).

9. Workineh Y, Semachew A, Ayalew E, Animaw W, Tirfie M, Birhanu M. Prevalence of perinatal asphyxia in East and Central Africa: systematic review and meta-analysis. Heliyon. 2020;6(4):e03793.

10. Wosenu L, Worku AG, Teshome DF, Gelagay AA. Determinants of birth asphyxia among live birth newborns in University of Gondar referral hospital, northwest Ethiopia: A case-control study. PloS one. 2018;13(9):e0203763.

11. Tasew H, Zemicheal M, Teklay G, Mariye T, Ayele E. Risk factors of birth asphyxia among newborns in public hospitals of Central Zone, Tigray, Ethiopia 2018. BMC research notes. 2018;11(1):496.

12. Woday A, Muluneh A, St Denis C. Birth asphyxia and its associated factors among newborns in public hospital, northeast Amhara, Ethiopia. PloS one. 2019;14(12):e0226891.

13. Aslam HM, Saleem S, Afzal R, Iqbal U, Saleem SM, Shaikh MW, et al. "Risk factors of birth asphyxia". Ital J Pediatr. 2014;40:94.

14. Berhe AK, Kassa GM, Fekadu GA, Muche AA. Prevalence of hypertensive disorders of pregnancy in Ethiopia: a systemic review and meta-analysis. BMC pregnancy and childbirth. 2018;18(1):34.

15. Dibaba B, Edosa D, Hajure M, Gebre G. Risk Factors of Antepartum Hemorrhage Among Mothers Who Gave Birth at Suhul General Hospital, 2016: A Case-Control Study. J Multidiscip Healthc. 2021;2021(14):271-278.

16. Antonucci R PA, Pilloni MD editor Perinatal asphyxia in the term newborn. The role of the clinical pathological dialogue in problem solving; 2014; Cagliari, Italy. Cagliari, Italy: JPNIM; 2014.

17. Haan M WJ, Khadem FV, Roth S, Gadian D, Mishkin M Brain and cognitive-behavioural development after asphyxia at term birth. Dev Sci. 2006;9(4):350-358.

18. Tette EMA, Nartey ET, Nuertey BD, Azusong EA, Akaateba D, Yirifere J, et al. The pattern of neonatal admissions and mortality at a regional and district hospital in the Upper West Region of Ghana; a cross sectional study. PloS one. 2020;15(5):e0232406.

19. Suleiman BM, Mokuolu OA, Adesiyun OO, Adeniyi A. Pattern of perinatal mortality in babies delivered at the university of ilorin teaching hospital, ilorin, Nigeria. West African journal of medicine. 2012;31(2):102-108.

20. Maternal and Child Health Directorate FMoH. National Strategy for Newborn and Child Survival in Ethiopia. Addis Ababa, Ethiopia: Federal Ministry of Health of Ethiopia; 2015. p. 10-18.

21. Tekelab T, Akibu M, Tagesse N, Tilhaun T, Yohanes Y, Nepal S. Neonatal mortality in Ethiopia: a protocol for systematic review and meta-analysis. Systematic Reviews. 2019;8(1):103.

22. Ethiopian Public Health Institute (EPHI)[Ethiopia] and ICF. Ethiopia Mini Demographic and Health Survey. Key Indicators. Rockville, Maryland, USA2019. p. 11-15.

23. Gebreheat G, Tsegay T, Kiros D, Teame H, Etsay N, Welu G, et al. Prevalence and Associated Factors of Perinatal Asphyxia among Neonates in General Hospitals of Tigray, Ethiopia, 2018. BioMed research international. 2018;2018:7.

24. Alemu A, Melaku G, Abera GB, Damte A. Prevalence and associated factors of perinatal asphyxia among newborns in Dilla University referral hospital, Southern Ethiopia- 2017. Pediatric Health Med Ther. 2019;10:69-74.

25. FDRE Ministry of Health. National Reproductive Health Strategy. Addis Ababa, Ethiopia: Ministry of Health; 2016. p. 100.

26. Silte Zone Health department: 2019/20 Fiscal Year Annual performance report of Silte Zone health Bureau. SNNPR In.; November, 2021.

27. Federal Ministry of Health. Neonatal Intensive Care Unit (NICU) Training. Management Protocol. Addis Ababa2014. p. 137.

28. Ibrahim NA, Muhye A, Abdulie S. Prevalence of Birth Asphyxia and Associated Factors among Neonates Delivered in Dilchora Referral Hospital, in Dire Dawa, Eastern Ethiopia. Clinics in Mother and Child Health. 2017;14(4).

29. Selvakumar R VC, Deepthy SIJ Incidence, Severity and Early Outcome of Hypoxicischemic Encephalopathy among Newborns Born in a Rural Tertiary Care Centre in Southern India. International Journal of Scientific Study. 2017;5(8):63-66.

30. Berglund S, Grunewald C, Pettersson H, Cnattingius S. Risk factors for asphyxia associated with substandard care during labor. Acta Obstet Gynecol Scand. 2010;89(1):39-48.

31. Wood S, Crawford S, Hicks M, Mohammad K. Hospital-related, maternal, and fetal risk factors for neonatal asphyxia and moderate or severe hypoxic-ischemic encephalopathy: a retrospective cohort study. J Matern Fetal Neonatal Med. 2019:1-6.

32. Ekwochi U, Asinobi NI, Osuorah CD, Ndu IK, Ifediora C, Amadi OF, et al. Incidence and Predictors of Mortality Among Newborns With Perinatal Asphyxia: A 4-Year Prospective Study of Newborns Delivered in Health Care Facilities in Enugu, South-East Nigeria. Clin Med Insights Pediatr. 2017;11:1-10.

33. Solayman M HS, Akber T , Islam MI , Islam MA. Prevalence of Perinatal Asphyxia with Evaluation of Associated Risk Factors in a Rural Tertiary Level Hospital. KYAMC Journal. 2017;8(1):43-48.

34. Samba A. Birth Asphyxia among Term Neonates at Korle-Bu Teaching Hospital (KBTH) in Accra. Obstetrics & Gynecology International Journal. 2017;7(6):1-4.

35. Gudayu TW. Proportion and factors associated with low fifth minute Apgar score among singleton newborn babies in Gondar University referral hospital; North West Ethiopia. African health sciences. 2017;17(1):1-6.

36. Abdo RA, Halil HM, Kebede BA, Anshebo AA, Gejo NG. Prevalence and contributing factors of birth asphyxia among the neonates delivered at Nigist Eleni Mohammed memorial teaching hospital, Southern Ethiopia: a cross-sectional study. BMC pregnancy and childbirth. 2019;19(1):536.

37. Nguanboonmak A SS. Prevalence and Risk Factors of Birth Asphyxia among Elderly Gravidarum. Thai Jour of Obst and Gynae. 2019;27(1):29-37.

38. Ilah B, Aminu M, Musa A, Adelakun M, Adeniji A, Kolawole T. Prevalence and Risk Factors for Perinatal Asphyxia as Seen at a Specialist Hospital in Gusau, Nigeria. Sub-Saharan African Journal of Medicine. 2015;2(2):64-68.

39. Royal College of Obstetricians and Gynaecologists. Antepartum Haemorrhage Green–top Guideline. UK: RCOG; 2011. p. 1-15.

40. Maternal and perinatal outcome of antepartum hemorrhage at three teaching hospitals in Addis Ababa, Ethiopia. Ethiopian Journal of Reproductive Health. 2020;12(3):1-19.

41. Utomo MT E, Winardi B Maternal Risk Factors Of Premature Delivery In Dr Soetomo Hospital. Folia Medica Indonesiana. 2011;47(1):1-45.

42. Woticha EW, Deressa W, Reja A. Barriers for detection and management of gestational diabetes mellitus in southern Ethiopia: A qualitative study. Diabetes Metab Syndr. 2019;13(3):1827-1831.

43. Muche AA, Olayemi OO, Gete YK. Gestational diabetes mellitus increased the risk of adverse neonatal outcomes: A prospective cohort study in Northwest Ethiopia. Midwifery. 2020;87:1-9.

44. Kiyani AN KA, Ehsan A Perinatal Factors Leading to Birth Asphyxia among Term Newborns in a Tertiary Care Hospital. Iran J Pediatr. 2014;24(5):637-642.

45. Mitao M, Philemon R, Obure J, Mmbaga BT, Msuya S, Mahande MJ. Risk factors and adverse perinatal outcome associated with low birth weight in Northern Tanzania: a registry-based retrospective cohort study. Asian Pacific Journal of Reproduction. 2016;5(1):75-79.

46. Borna S, Borna H, Khazardoost S, Hantoushzadeh S. 'Perinatal outcome in preterm premature rupture of membranes with Amniotic fluid index < 5 (AFI < 5). BMC pregnancy and childbirth. 2004;4(1):15.

47. Tewesa E, Chirwa E, Majamanda MD, Maluwa A, Chimwaza A. Associative Factors for Birth Asphyxia at Queen Elizabeth Central Hospital—Malawi. Journal of Biosciences and Medicines. 2017;05(05):22-31.

48. Mehta V, E A, Spoorthi, Archana, Hassan M. Meconium-stained amniotic fluid as a risk factor for perinatal asphyxia. International Journal of Contemporary Pediatrics. 2019;6(2).

49. Gebregziabher GT, Hadgu FB, Abebe HT. Prevalence and Associated Factors of Perinatal Asphyxia in Neonates Admitted to Ayder Comprehensive Specialized Hospital, Northern Ethiopia: A Cross-Sectional Study. Int J Pediatr. 2020;2020:1-8.

50. Engida Yisma1 BD, Ayalew Astatkie, Nebreed Fesseha. Knowledge and utilization of partograph among obstetric care givers in public health institutions of Addis Ababa, Ethiopia. BMC Pregnancy & Childbirth. 2013;13(17):1-9.

51. Hagos AA, Teka EC, Degu G. Utilization of Partograph and its associated factors among midwives working in public health institutions, Addis Ababa City Administration,Ethiopia,2017. BMC pregnancy and childbirth. 2020;20(1):49.

52. Haile Y, Tafese F, Weldemarium TD, Rad MH. Partograph Utilization and Associated Factors among Obstetric Care Providers at Public Health Facilities in Hadiya Zone, Southern Ethiopia. J Pregnancy. 2020;2020:8.

53. Markos M, Arba A, Paulos K. Partograph Utilization and Associated Factors among Obstetric Care Providers Working in Public Health Facilities of Wolaita Zone, 2017. J Pregnancy. 2020;2020:8.

54. Wakgari N, Amano A, Berta M, Tessema GA. Partograph utilization and associated factors among obstetric care providers in North Shoa Zone, Central Ethiopia: a cross sectional study. African health sciences. 2015;15(2):552-559.

Perinatal asphyxia and associated factors among neonates admitted to a specialized public hospital in South Central Ethiopia: A retrospective cross sectional study

Seifu Awgchew Mamo [1¶], Girum Sebsibie Teshome [2¶], Tewodros Tesfaye[2¶], Abel Tibebu Goshu[3¶*]

[1]Department of Pediatric Nursing, College of Medicine and Health sciences, Wachemo University, Hosaena, Ethiopia

[2]School of Nursing and Midwifery, College of Health Sciences, Addis Ababa University, Addis Ababa, Ethiopia

[3]School of Nursing and Midwifery, College of Health and Medical sciences, Haramaya University, Harar, Ethiopia

*Corresponding Author

Abel Tibebu Goshu

Email: ABTI2222@GMAIL.COM

¶ - These authors contributed to this work equally.

Abstract

Introduction: Perinatal asphyxia ~~in the world~~ continues to be a significant clinical concern around the world as the consequences can be devastating. World Health Organization data indicates perinatal asphyxia is encountered amongst 6 - 10 newborns per 1000 live full-term birth, and the figures are higher for low and middle-income countries. Nevertheless, studies on the prevalence of asphyxia and~~in~~ the extent of the problem in poorly resourced southern Ethiopian regions.~~these countries~~ are limited. This study is aimed to determine the magnitude of perinatal asphyxia and its associated factors. **Methods:** A retrospective cross-sectional study design was used from March to April 2020. Data was collected from charts of neonates who were admitted to NICU from January, 2016 to December 31, 2019. **Result:** The review of 311 neonates' medical records revealed that 41.2% of the neonates experienced perinatal asphyxia. Preeclampsia during pregnancy (AOR= 6.2, 95%CI :3.1- 12.3), antepartum hemorrhage (AOR= 4.5, 95%CI:2.3- 8.6), gestational diabetes mellitus (AOR= 4.2, 95%CI:1.9-9.2), premature rupture of membrane (AOR= 2.5, 95%CI:1.33-4.7) fetal distress (AOR= 3,95%CI:1.3-7.0) and meconium-stained amniotic fluid (AOR= 7.7, 95%CI : 3.1-19.3) were the associated factors. **Conclusion:** Substantial percentages of neonates encounter perinatal asphyxia, causing significant morbidity and mortality. Focus on early identification and timely treatment of perinatal asphyxia in hospitals should, therefore, be given priority.

Keywords: Neonate, Perinatal asphyxia, NICU, Worabe, Ethiopia

Introduction

A decade remains to achieve the third Sustainable Development Goal (SDG) targets, including reducing preventable causes of neonatal mortalities (1, 2). Though the number of neonatal deaths has declined significantly within the last thirty years, Africa alone accounted for about 41% of the total global neonatal mortality in the year 2017 (3).

Perinatal asphyxia, also called birth asphyxia, is one of the leading causes of neonatal deaths in the world following severe infections and prematurity (1, 4). It results from the loss of the blood supply or impairment of gas exchange to or from the fetus before, during, or after the birth process (5). Perinatal asphyxia may lead to severe metabolic acidosis, hypercarbia, progressive hypoxemia, neonatal encephalopathy, and multi-system organ failure, and even result in death (6-10).

An arterial blood sample, APGAR score (appearance, pulse, grimaces, activity, and respiration), immediate neurological complications or an evidence of multiple organ dysfunction are used to diagnose asphyxia. The APGAR score is assessed in the first and fifth minutes of life and ranges from zero to ten. According to the WHO classification, an APGAR score of four to seven in the first minute of life indicates moderate prenatal asphyxia, whereas a score of zero to three suggests severe asphyxia(11, 12).

Factors related to antepartum, intrapartum or immediate postpartum period may contribute to perinatal asphyxia development (13). These could be summarized into abnormal maternal oxygenation, congenital infections, insufficient placental perfusion, traumatic deliveries, or impaired umbilical circulation. Most studies identified that intrapartum factors as having the highest impact (7, 14, 15).

Worldwide, perinatal asphyxia is encountered amongst 6 - 10 newborns per 1000 live full-term birth (9, 16, 17). Apparently, the numbers are higher for low and middle-income countries. In Africa, significant neonatal morbidity and mortality occur due to complications associated with perinatal asphyxia. Studies from different regions of the continent show variant figures on the magnitude. A study from two district hospitals from Ghana showed that birth asphyxia to be the second cause of admission (15.1%) and the third cause of mortality (20.7%). In contrast, another study conducted in Nigeria indicated severe perinatal asphyxia to be the most important cause of death in all birth weight categories except in extremely low

birth weight babies (18, 19). A systematic review conducted amongst east and central African countries has shown a pooled prevalence of perinatal asphyxia to be 15.9% (9).

Ethiopian studies are no different from the rest of Africa. Though the country has made efforts to minimize under five and neonatal mortality rates by devising a National strategy for Newborn and Child Survival, current figures still show demand for committed action to target and tackle preventable causes like birth asphyxia (20, 21). The Ethiopian Demographic and Health survey in 2019 showed a reduction in early childhood mortality except for neonatal mortality, which had steady progress since 2016. It was shown that birth asphyxia was and still is the primary cause of neonatal deaths (22). Observational studies in different parts of the country also magnify this concern. A cross-sectional study conducted in the Tigray region of northern Ethiopia showed that 22.1% of the total neonates included in the study experienced perinatal asphyxia (23). At the same time, another study from Dilla referral hospital revealed the prevalence of perinatal asphyxia to be 32.8% (24).

In order to improve the quality of care that is delivered within a country, one must first understand the problem and the issues that healthcare providers encounter. Improving the care of the maternal-infant dyad can lead to a significant decrease in the rates of perinatal asphyxia (22, 25). In an effort to enhance care in Ethiopia, we sought to understand the burden of perinatal asphyxia and early assessment practices. To do this, we evaluated cases of perinatal asphyxia and identified associated factors among neonates admitted to the neonatal intensive care unit of Worabe Comprehensive Specialized Hospital (WCSH) in southern Ethiopia.

Methods

Study area and period

The study was conducted at Worabe Comprehensive Specialized Hospital (WCSH), Silte zone, south central Ethiopia, from March to April 2020 (by reviewing neonates' charts from January/2016 to December 31/2019). WCSH was established in October 2014 and is located 170 km southwest of Addis Ababa. It is the only referral hospital in Silte zone and provides emergency, out-patient and in-patient services to the

local and neighboring communities. The Neonatal Intensive Care Unit has 18 beds, 5 incubators, 15 radiant warmers and operates with 17 nurses, 2 midwives, 1 general practitioner, 2 pediatricians and 1 gynecologist. The NICU offers diagnostic and treatment services for approximately 1000-1500 newborns per year (26). The level of newborn care is determined by the neonate's gestational age, birth weight, sickness severity, and the facility's general setup. As a result, health practitioners at level I should be able to offer basic newborn care to low-risk infants and triage unwell newborns. Specialty treatment (Level II NICU) is confined to newborn infants over 32 weeks gestational age weighing 1500 g or recovering from serious disease treated in a level III environment (subspecialty). All newborn infants with extreme preterm (28 weeks or less) or extremely low birth weight (1000 g or less) or severe and/or complex disease are handled in level III NICU (26).

Study design

A facility-based retrospective cross-sectional study design was employed.

Population

Source population

All records' of neonates who were admitted at NICU of Worabe comprehensive specialized hospital~~-~~. ~~from January /2016 to December 31/2019~~

Study population

All the records' of neonates who ~~fulfilled the inclusion criteria and~~ were admitted to the NICU of WCSH were added to the study. The records' of neonates with incomplete documentation (which has no proper maternal or fetal measurement parameters), with major congenital malformations or anomalies were excluded.

~~Eligibility criteria~~

~~Inclusion criteria~~

~~Records' of all neonates admitted at NICU of WCSH from January /2016 to December 31/2019.~~

~~Exclusion criteria~~

~~Records of neonates with incomplete documentation (which has no proper maternal or fetal measurement parameters), with major congenital malformations or anomalies~~

Sample size determination

The sample size was calculated using the single population proportion formula considering the p-value of 32.8% from a previous study conducted in Ethiopia (24)

$$ni = \frac{(z\alpha/2)^2 * p\,(1 - p)}{d^2}$$

Where, ni = initial sample size, p = proportion of prenatal asphyxia; 32.8% = 0.328, $(z\alpha/2)^2$= confidence interval (95%), d= is the margin of sampling error tolerated (5%) = 0.05

Substituting the values for each of these variables in the above formula, the sample size was estimated to be 339.

Because the source population is less than ten thousand, we used a correction formula

$$nf = ni/\ (1+ni/N)$$

Where nf= final sample size

ni = calculated sample size (initial sample size)

N= total number of neonates admitted to NICU of WCSH within the three years period (3796 neonates)

$$nf = 339/(1+339/3796) = 311$$

Sampling procedure

Systematic random sampling technique was used to select which charts to review. After the card

records of the neonates were put in their order of admission, the kth interval was determined by dividing the total population size by the total sample size.

$$k = N/nf=3796/311=12$$

Where, N is the total population at NICU of WCSH from January /2016 to December 31/2019,

nf = final sample size of the study).

Operational definitions

Perinatal asphyxia – is the inability of a newborn to initiate and sustain respiration by persistently scoring an APGAR score of less than 7 for more than 5 minutes after delivery (24, 27).

Prolonged labor – is the first stage of labor, exceeding 12 hours in primigravida or 8 hours in multipara mothers (25).

Premature rupture of membranes (PROM) – rupture of the amniotic sac and chorion membrane occurred before the onset of labor (24).

Study variables

Magnitude of perinatal asphyxia was the outcome variable. Maternal socio-demographic variable (age in year, educational status, religion, residency, and occupation), antepartum related variable (anemia during Pregnancy, ANC follow up, DM, pregnancy-induced hypertension, antepartum hemorrhage, chronic hypertension, gravidity, and parity), intrapartum related variable (PROM, prolonged labor, obstructed labor, place of delivery and mode of delivery), neonatal related variable (birth weight, fetal distress, the gender of the neonate and intrauterine meconium release) were independent variables.

Data collection tools and procedures

Data was collected from neonates' registration and medical records using a structured checklist adapted and modified from different literature(13, 23, 24, 28). The checklist contains four sections which assess socio-

demographic, antepartum factors (parity/gravidity, gestational age, complications/illnesses during pregnancy), intrapartum factors (mode of delivery, duration of labor, fetal presentation, fetal distress, intrauterine meconium release occurrence of PROM, and obstructed labor) and neonatal factors (sex, birth weight, gestational age at birth and occurrence of perinatal asphyxia). To identify maternal-related factors, the maternal record chart of the same year was traced. Three BSc nurses were recruited as data collectors and were trained for two days on the checklist, data collection, and supervision. Furthermore, the sampling procedures were also elucidated to them. The checklist was pretested for its relevance and clarity to answer the research question.

Ethical Considerations

Ethical clearance for the commencement of the study was obtained from the Addis Ababa University, School of Nursing and Midwifery Research Ethical Committee (Ref. no. 030/20/SNM). Permission was also sought and obtained from the ethical committee of Worabe comprehensive specialized hospital. Data was kept anonymous by keeping the identity of the neonate's or mother's credentials hidden before, during, and after the study.

Data analysis and management

The data were entered into statistical software Epi-data version 3.1. The entered data was subject to cleaning using simple frequency and tabulation to ensure the data's validity. Then the analysis was made with IBM SPSS version 24.0 after exporting the prepared data. Descriptive statistics, such as frequency distribution, were computed to describe the significant variables of the study. Odds ratio and the p-value were computed. $P \leq 0.05$ was considered as statistically significant for association. Binary logistic regression was conducted to see the effect of each of the independent variables on the outcome variable, and variables that were statistically significant at P value less than 0.05 levels were put into the final model (multivariate analysis) to control for confounding.

Results

Socio-demographic characteristics of the mothers' of neonates

Data were collected from a total of 311 neonatal medical records. Of the neonates' mothers, 105(33.8%) were between the ages of 25-29 years, and 163(52.4%) of the mothers had no formal education. Almost all the mothers, 297 (95.5%), were Muslim by religion **(Table 1).**

Table.1 Socio-demographic characteristic of mothers of newborns at WCSH, South central Ethiopia (n=311)

Variable	Category	Frequency/ Percentage of mothers' of neonates (n=311)	Frequency/Percentage of mothers' of asphyxiated neonates (n=128)
Age	≤19yr	12 (3.9%)	6 (4.68%)
	20-24yr	65 (20.9)	24 (18.75%)
	25-29yr	105 (33.8%)	44 (34.37%)
	30-34yr	84 (27%)	34 (26.56%)
	≥35	45 (14.5%)	20 (15.625%)
Educational status	No formal education	163 (52.4%)	78 (60.93%)
	Primary	64 (20.6%)	24 (18.75%)
	Secondary	45 (14.5%)	14 (10.93%)
	Higher education	39 (12.5%)	12 (9.37%)
Residence	Rural	207 (66.6%)	93 (72.65%)
	Urban	104 (33.4%)	35 (27.34%)

Religion	Muslim	297 (95.5%)	123 (96%)
	Orthodox	10 (3.2%)	3 (2.34%)
	Protestant	4 (1.3%)	2 (111.56%)
	Catholic	0 (0%)	0 (0%)
Occupation status	House wife	120 (38.6%)	57 (44.53%)
	Private	2 (0.6%)	1 (0.78%)
	Government	63 (20.3%)	23 (17.96%)
	merchant	112 (36%)	46 (35.93%)
	Student	14 (4.5%)	1 (0.78%)

Obstetrics history of the mothers at WCSH

Regarding parity, 196(63%) mothers were multiparous, and 197(63.3%) of them were attending their antenatal follow up. Among the mothers, 129(41.5%) had anemia, while 86(27.7%) were diagnosed with preeclampsia. Only 61(19.6%) of mothers had gestational diabetes mellitus, and few mothers, 21(6.8%), had chronic hypertension. Of the 311 mothers, 92(29.6%) had an antepartum hemorrhage, and 222(71.4%) mothers of the neonates delivered by SVD. Concerning membrane status, 100(32.2%) mothers developed premature rupture of the membrane, and 115(37%) mothers had obstructed labor. **(Table 2)**

Table-2 Obstetrics history of mothers at WCSH, South central Ethiopia (n=311)

Variable	Category	Frequency(n=311)	Percentage (%)
Parity	Primipara	115	37%
	Multipara	196	63%
Gestational age	<37weeks	43	13.8%
	>42weeks	18	5.8%
	37week-42week	250	80.4%
ANC[a] follow up	Yes	197	63.3%
	No	114	36.7%
Anemia	Yes	129	41.5%
	No	182	58.5%
Preeclampsia/eclampsia	Yes	86	27.7%
	No	225	72.3%
Chronic hypertension	Yes	21	6.8%

	No	290	93.2%
APH[b]	Yes<br>No	92<br>219	29.6%<br>70.4%
DM	Yes<br>No	24<br>287	7.7%<br>92.3%
Gestational DM[c]	Yes<br>No	61<br>250	19.6%<br>80.4%
Chronic illness	Yes<br>No	247<br>64	79.4%<br>20.6%
Mode of delivery	SVD[d]<br>C/S[e]<br>Instrumental	222<br>72<br>17	71.4%<br>23.2%<br>5.5%
Duration of labor	>12hr(prolong)<br><12hr	95<br>216	30.5%<br>69.5%
PROM[f]	Yes<br>No	100<br>211	32.2%<br>67.8%
Obstructed labor	Yes<br>No	115<br>196	37%<br>63%

[a]Antenatal Care, [b]Antepartum Hemorrhage, [c]Diabetes mellitus, [d]Spontaneous Vaginal Delivery, [e]Cesearian Section, [f]Prematue Rupture of Membrane

Clinical characteristics of neonates at WCSH

From the total number of neonates, 192(61.7%) of them were males, and 119 (38.3%) were females, and most of the neonates, 240(77.2%), had birth weight between 2.5kg to 4kg. Only 84(27%) of babies passed their meconium during labor, and 119(38.3%) of them developed fetal distress. ~~The overall prevalence of perinatal asphyxia was 128(41.2%)~~ **(Table 3)**~~.~~

Table-3 Clinical characteristics of neonates at WCSH, South central Ethiopia (n=311)

**Characteristics**	**Category**	**Frequency(n=311)**	**Percentage (%)**
Presentation	Vertex<br>Non vertex	251<br>60	80.7%<br>19.3%
Place of delivery	Home	10	3.2%

	Health center	157	50.5%
	Private clinic	3	1%
	Hospital	141	45.5 %
Gender	Male	192	61.7%
	Female	119	38.3%
Birth weight	<2.5kg	59	19%
	2.5kg-4kg	240	77.2%
	>4kg	12	3.9%
Intrauterine meconium release	Yes	84	27%
	No	227	73%
Fetal distress	Yes	119	38.3%
	No	192	61.7%
Perinatal asphyxia	Yes	128	41.2%
	No	183	58.8%

Magnitude of perinatal asphyxia

The overall prevalence of perinatal asphyxia among neonates who were delivered at WCHS was found to be 128(41.2%).

Factors associated with the occurrence of perinatal asphyxia

Different socio-demographic, neonatal, and maternal variables were tested for their association with the presence of perinatal asphyxia. For the multivariate logistic regression, all variables that were found to have an association with the outcome variable in bivariate logistic regression at P=0.25 were included in the multivariate logistic regression models. During multivariate logistic regression, six variables (preeclampsia, antepartum hemorrhage, gestational diabetes mellitus, premature rupture of membrane, intrauterine meconium release, and fetal distress during labor) were found significantly associated with perinatal asphyxia (at $p \leq 0.05$).

Among maternal factors, preeclampsia was independently associated with significantly higher odds of the newborn developing perinatal asphyxia (AOR= 6.2, 95% CI, 3.1, 12.3). Neonates from mothers who had antepartum hemorrhage were 4.5 times more affected by asphyxia as compared to neonates who delivered from mothers who had no antepartum hemorrhage(AOR= 4.5, 95% CI, 2.3, 8.6). Furthermore, neonates from mothers with gestational diabetes mellitus were four times more likely to be asphyxiated than neonates from mothers without gestational diabetes mellitus (AOR= 4.2, 95% CI 1.9,9.2). Moreover, neonates of mothers who had a history of premature rupture of the membrane were 2.5 times more likely to be asphyxiated than those who had not encountered premature rupture (AOR= 2.5, 95%CI,1.33,4.7). Among the fetal factors, fetal distress during labor and meconium-stained amniotic fluid were significantly associated with asphyxia (AOR= 3, 95%CI, 1.3.7.0) and (AOR= 7.7, 95%CI, 3.1, 19.3) respectively. **(Table 4)**

Table-4 Factors associated with perinatal asphyxia among neonates admitted at WCSH, South central Ethiopia (n=311)

Variables	Asphyxiated		COR 95%CI	AOR 95%CI	p-value
	No	Yes			
**Educational status**					
No formal education	85	78	2.07(.98, 4.4)	1.6(.5, 5.1)	0.420
Primary	40	24	1.4(.6, 3.2)	1.2(.4, 3.7)	0.624
Secondary	31	14	1.0(.4, 2.6)	1.2(.4, 3.5)	0.607
Higher education	27	12	1	1	
**Residence**					
Rural	114	93	1.6(.985,2.63)	1.0(0.4,2.3)	0.911
Urban	69	35	1	1	
**Preeclampsia/Eclampisa**					
Yes	**25**	**61**	**5.75(3.33, 9.93)**	**6.2(3.1,12.3)**	**0.000***
No	158	67	1	1	
**Antepartum hemorrhage**					
Yes	**31**	**61**	**4.464 (2.656, 7.5)**	**4.5(2.3,8.6)**	**0.000***

No	152	67	1	1	
**Gestational diabetes mellitus**					
Yes	**17**	**44**	**5.115(2.757,9.5)**	**4.2(1.9,9.2)**	**0.000***
No	166	84	1	1	
**Mode of delivery**					
SVD[a]	139	83	1	1	
CS[b]	39	33	1.417(.828,2.425)	1.0(0.5,2)	0.912
Instrumental	5	12	4.019(1.368,11.81)	2.3(0.5,9.5)	0.258
**Duration of labor**					
>12hr	49	46	1.534(.942,2.5)	1.1(0.6,2.2)	0 .703
<12hr	134	82	1	1	
**Presentation of the fetus**					
Vertex	153	98	1	1	
Non vertex	30	30	1.561(.886,2.75)	1.5(0.7,3.5)	0.311
**Premature rupture of membrane**					
Yes	**42**	**58**	**2.782(1.705,4.54)**	**2.5(1.33,4.7)**	**0.004***
No	141	70	1	1	
**Obstructed labor**					
Yes	52	63	2.442(1.522,3.92)	1.9(1.0,3.8)	0.053
No	131	65	1	1	
**Fetal distress during labor**					
Yes	**59**	**60**	**1.854(1.164,2.95)**	**3.0(1.3, 7.0)**	**0.010***
No	124	68	1	1	
**Intrauterine meconium release**					
Yes	**23**	**61**	**6.33(3.62,11)**	**7.7(3.1,19.3)**	**0.000***
No	160	67	1	1	
**Birth weight**					
< 2.5kg	40	19	1	1	
2.5kg-4kg	137	103	2.105(0.6, 7.39)	2.67(0.6, 12.6)	0.207
>4kg	6	6	1.33(.417, 4.24)	2.904(0.7,12.2)	0.146

[a]Spontaneous Vaginal Delivery, [b]Cesearian Section, *AOR= statistically significant at $p \leq 0.05$

Discussion

This study investigated the magnitude of perinatal asphyxia and associated factors among neonates admitted at the neonatal intensive care unit of Worabe comprehensive specialized hospital.

The findings from this study revealed that the magnitude of perinatal asphyxia was 41.2%, which is quite higher compared to a study done in southern India (2.7%)(29), Sweden (5.4%) (30), Alberta Canada (2.28%)(31) and South-East Nigeria (12.8%) (32). This discrepancy could be due to the explained by the socioeconomic variation between the study area and the other countries. Better developed countries have the necessary infrastructure and skilled health care providers to significantly reduce the incidence of perinatal complications. Nonetheless, the finding is lower than a study done in Bangladesh (56.9%) (33) and Ghana 283(61.8%) (34). The possible explanation for this variation stems from the latter two studies being conducted in poorly resourced areas. Additionally, the higher magnitude in these studies may be due to the reported low number of healthcare professionals trained to conduct neonatal resuscitation. Comparably, this study's result was higher than other studies conducted elsewhere in Ethiopia (12, 23, 28, 35, 36). The difference could be attributed to the relative urban nature of the cities, which in turn affects the quality of setup amongst the facilities, the living standards of resident mothers and accessibility of health institutions. This is a critical indicator that the case of perinatal asphyxia needs more emphasis in the study area.

The Ethiopian ministry of health adopted the WHO recommendation of at least four ANC follow-up visits throughout a pregnancy. Despite this, ANC is utilized well in urbanized cities like Addis Ababa and Dire Dawa while semi urban and rural areas are far behind (22). This is attributed to maternal illiteracy and low socio-economic status in the latter as these factors have direct relations to the delay in health seeking behavior(25). The non-adherence to follow up could be catastrophic as it could endanger the maternal and fetal outcomes. This study revealed that 36.7% of mothers of neonates with asphyxia were not strictly attending their antenatal follow-up, paving the way for the 'silent' progress of pregnancy-induced complications (PICs) and associated perinatal outcomes.

A sum of factors could attribute to the devastating outcomes of PICs. The unpredictable nature of PICsthese conditions, poor obstetric care and low service utilizations in Ethiopia, largely give rise to the maternal and perinatal sequelae. Even though the country adopted the “three delays” model;– a set of strategies targeting delays in decision to seek care, delays in seeking care and delays in receiving adequate health careto target and resolve the burden of PICs; successful implementation yet needs significant work (25). Such pitfalls are reflected through undesired outcomes like perinatal asphyxia. This e present study also has identified major PICs including preeclampsia, antepartum hemorrhage, gestational diabetes mellitus, premature rupture of the membrane continue to be significant predictors of perinatal asphyxia.

Preeclampsia and eclampsia are known predictors of maternal and perinatal morbidities and mortalities (14). These disorders, categorized under Pregnancy Induced Hypertensive Disorders (PIHD) are common concerns globally, even more in low income countries like Ethiopia. Studies have shown that variations in prevalence of PIHDs can be observed within a country. For instance, the SNNPR region where the study area is located is one of the poorly resourced areas of the country and has a higher percentage of PIHD than any the other region in the country (14). Despite the absence of novel approaches to exactly predict the occurrence of preeclampsia, complications like birth asphyxia could be lowered through frequent and consistent follow-up during pregnancy.This study found out reaffirmed that preeclampsia is significantly associated with perinatal asphyxia. Studies conducted in India (29), Bangkok Rajavithi hospital (37), Gusau Nigeria (38), and Tigray Ethiopia (23) are in line with this data. Preeclampsia and eclampsia are known predictors of maternal and perinatal morbidities and mortalities (14). These disorders, categorized under Pregnancy Induced Hypertensive Disorders (PIHD) are common concerns globally, even more in low income countries like Ethiopia. Studies have shown that variations in prevalence of PIHDs can be observed within a country. For instance, the SNNPR region where the study area is located is one of the poorly resourced areas of the country and has a higher percentage of PIHD than any the other region in the country (14). Despite the absence of novel approaches to exactly predict the occurrence of preeclampsia, complications like birth asphyxia could be lowered through frequent and consistent followup during pregnancy. Furthermore, a favorable perinatal outcome is dependent on the availability of quality care within the appropriate time.

Significant evidence reveals (15, 39, 40) that the occurrence of antepartum bleeding results in a decreased blood flow from the mother to the placenta with subsequent hypoxia to the fetus. Studies conducted in Ethiopia have also shown an increased incidence of perinatal asphyxia among mothers who encountered APH (15, 40). ~~The incidence of APH by itself may not vary across the developed and developing countries. Though, the negative maternal and neonatal outcomes in the latter could be traced back to poor obstetric care~~ (15, 41, 42)~~.~~ This study also identified that antepartum hemorrhage was associated with perinatal asphyxia, consistent with the study conducted in Bangalore, Indonesia (43), and Accra Ghana (34).

Gestational diabetes mellitus was also a factor associated with perinatal asphyxia, and this result is in agreement with what was reported in Canada (31), Pakistan (13), and the study done in Sweden (30). The possible explanations for these are the metabolic derangement responsible for the inadequate production of surfactants. In Ethiopia, health care providers screen and initiate counseling for lifestyle modification during the early stages of the diagnosis, followed by a prescribed insulin regimen. Nevertheless, ~~this~~ effective implementation of these strategies once again is hindered ~~by lack of adequate followup by mothers, insufficiently equipped setup and poorly skilled health care providers~~ (44, 45). ~~Inability to get lifesaving emergency care to treat impending complications also adds to the burden.~~

The current ~~is~~ study ~~also~~ found out that premature rupture of the membrane was significantly associated with perinatal asphyxia~~; this is~~ which is consistent with other findings from~~; studies conducted in~~ Pakistan (30, 46), Ghana (34), and Tanzania (47). The ~~rationale for this~~ similarity between the studies may be explained by the scientific evidence that links ~~linked~~ PROM to the incidence of oligohydramnios, resulting in possible infection and umbilical cord compression (48). ~~Screening for infections during follow-up visits is the most feasible strategy to significantly minimize the risk of perinatal asphyxia resulting from PROM. Factors such as fetal distress, intrauterine meconium release and aspiration have been known to significantly impact the perinatal outcome. PICs and additional fetal conditions are evidenced to be the major causes. These conditions solely or in combination could effect in short and long term complications like perinatal asphyxia.~~

The present study identified fetal distress and meconium-stained amniotic fluid as significant factors impacting the occurrence of perinatal asphyxia. Comparable results were found from other parts of the world; Malawi (49), Thailand(37), Pakistan(46), and India(50). Studies conducted in central, south, and northern parts of Ethiopia are also in line with this (24, 36, 51). The rationale for the poor perinatal outcome in Ethiopia likely stems from sub-standard partograph utilization, poor intrapartum care, and ineffective neonatal resuscitation practices (41, 52-55).

Limitations

Eventhough the study generated pertinent data that can be used as a baseline, the results can only describe the context in ~~Silte zone and~~ WCSH. Furthermore the study also did not include other health lower health care facilities where deliveries are conducted. The hospital where the study was conducted is an inistitution where pregnant women with complications are referred to, and this may have inflated the magnitude of the problem. This study utilized only the APGAR score to diagnose perinatal asphyxia. Additional investigations such as the arterial blood gas analysis for pH were not considered. ~~Utilization of APGAR score may not be conclusive as superior investigation techniques like arterial blood gas analysis are better in diagnosis.~~ The nature of ~~cross~~ cross-sectional study design ~~has also~~also has an impact in defining cause and effect relationships.

Conclusion

The study revealed that a substantial percentage of neonates experienced perinatal asphyxia. Major PICs such as preeclampsia, APH, and gestational DM, PROM, and fetal factors like fetal distress and intrauterine meconium release were significantly associated with the occurrence of perinatal asphyxia. Focus on early identification and timely treatment of perinatal asphyxia in hospitals should, therefore, be given priority. Effective implementation of community engaging approaches such as mobilization and sensitization would improve the utilization of ANC services among mothers. Furthermore, revitalizing maternal waiting homes in the area may mitigate the delays in seeking care and associated complications. The Ministry of health

and the hospital administrators need to devise strategies that strengthen the health care system, meliorate the obstetric care setup and ensure the adherence of health care providers to intrapartum and immediate postpartum protocols such as partograph utilization and newborn resuscitation., Researchers should focus on conducting implementation researches to assess the relative impact of strengthening and revitalizing of best practices.

Abbreviations: (SDG) Sustainable Development Goal, WHO (World Health Organization), WCSH (Worabe Comprehensive Specialized Hospital), SVD (Spontaneous Vaginal Delivery), ANC (Antenatal Care) NICU (Neonatal Intensive Care Unit), PROM (Premature Rupture of Membrane), DM (Diabetes Mellitus), CS (Cesarean Section), PICs (Pregnancy Induced Complications), PIHDs (Pregnancy Induced Hypertensive Disorders).

Acknowledgment

We are indebted to Worabe comprehensive specialized hospital for providing us permission to conduct the study.We would like to thank Addis Ababa University, College of Health Sciences, for the offered technical support. Finally, the authors are also thankful to the supervisors and data collectors.

Authors' contributions

All authors collaboratively made substantial contributions to conception and design, acquisition of data, or analysis and interpretation of data; took part in drafting the article or revising it critically for important intellectual content; gave final approval of the version to be published; and agree to be accountable for all aspects of the work.

Disclosure

The authors have no conflict of interest to disclose.

References

1. WHO. MCEE-WHO methods and data sources for child causes of death 2000-2016. Geneva, Switzerland2018.

2. WHO. Towards a global action plan for healthy lives and well-being for all World Health Organization; 2018. p. 37.

3. Hug L, Alexander M, You D, Alkema L. National, regional, and global levels and trends in neonatal mortality between 1990 and 2017, with scenario-based projections to 2030: a systematic analysis. The Lancet Global Health. 2019;7(6):e710-e720.

4. Oza S, Lawn JE, Hogan DR, Mathers C, Cousens SN. Neonatal cause-of-death estimates for the early and late neonatal periods for 194 countries: 2000-2013. Bull World Health Organ. 2015;93(1):19-28.

5. Goldenberg RL, Harrison MS, McClure EM. Stillbirths: The Hidden Birth Asphyxia - US and Global Perspectives. Clin Perinatol. 2016;43(3):439-453.

6. Allanson ER, Muller M, Pattinson RC. Causes of perinatal mortality and associated maternal complications in a South African province: challenges in predicting poor outcomes. BMC pregnancy and childbirth. 2015;15:37.

7. Simiyu IN, McHaile DN, Katsongeri K, Philemon RN, Msuya SE. Prevalence, severity and early outcomes of hypoxic ischemic encephalopathy among newborns at a tertiary hospital, in northern Tanzania. BMC pediatrics. 2017;17(1):131.

8. Usman F, Imam A, Farouk ZL, Dayyabu AL. Newborn Mortality in Sub-Saharan Africa: Why is Perinatal Asphyxia Still a Major Cause? Annals of global health. 2019;85(1).

9. Workineh Y, Semachew A, Ayalew E, Animaw W, Tirfie M, Birhanu M. Prevalence of perinatal asphyxia in East and Central Africa: systematic review and meta-analysis. Heliyon. 2020;6(4):e03793.

10. Wosenu L, Worku AG, Teshome DF, Gelagay AA. Determinants of birth asphyxia among live birth newborns in University of Gondar referral hospital, northwest Ethiopia: A case-control study. PloS one. 2018;13(9):e0203763.

11. Tasew H, Zemicheal M, Teklay G, Mariye T, Ayele E. Risk factors of birth asphyxia among newborns in public hospitals of Central Zone, Tigray, Ethiopia 2018. BMC research notes. 2018;11(1):496.

12. Woday A, Muluneh A, St Denis C. Birth asphyxia and its associated factors among newborns in public hospital, northeast Amhara, Ethiopia. PloS one. 2019;14(12):e0226891.

13. Aslam HM, Saleem S, Afzal R, Iqbal U, Saleem SM, Shaikh MW, et al. "Risk factors of birth asphyxia". Ital J Pediatr. 2014;40:94.

14. Berhe AK, Kassa GM, Fekadu GA, Muche AA. Prevalence of hypertensive disorders of pregnancy in Ethiopia: a systemic review and meta-analysis. BMC pregnancy and childbirth. 2018;18(1):34.

15. Dibaba B, Edosa D, Hajure M, Gebre G. Risk Factors of Antepartum Hemorrhage Among Mothers Who Gave Birth at Suhul General Hospital, 2016: A Case-Control Study. J Multidiscip Healthc. 2021;2021(14):271-278.

16. Antonucci R PA, Pilloni MD editor Perinatal asphyxia in the term newborn. The role of the clinical pathological dialogue in problem solving; 2014; Cagliari, Italy. Cagliari, Italy: JPNIM; 2014.

17. Haan M WJ, Khadem FV, Roth S, Gadian D, Mishkin M Brain and cognitive-behavioural development after asphyxia at term birth. Dev Sci. 2006;9(4):350-358.

18. Tette EMA, Nartey ET, Nuertey BD, Azusong EA, Akaateba D, Yirifere J, et al. The pattern of neonatal admissions and mortality at a regional and district hospital in the Upper West Region of Ghana; a cross sectional study. PloS one. 2020;15(5):e0232406.

19. Suleiman BM, Mokuolu OA, Adesiyun OO, Adeniyi A. Pattern of perinatal mortality in babies delivered at the university of ilorin teaching hospital, ilorin, Nigeria. West African journal of medicine. 2012;31(2):102-108.

20. Maternal and Child Health Directorate FMoH. National Strategy for Newborn and Child Survival in Ethiopia. Addis Ababa, Ethiopia: Federal Ministry of Health of Ethiopia; 2015. p. 10-18.

21. Tekelab T, Akibu M, Tagesse N, Tilhaun T, Yohanes Y, Nepal S. Neonatal mortality in Ethiopia: a protocol for systematic review and meta-analysis. Systematic Reviews. 2019;8(1):103.

22. Ethiopian Public Health Institute (EPHI)[Ethiopia] and ICF. Ethiopia Mini Demographic and Health Survey. Key Indicators. Rockville, Maryland, USA2019. p. 11-15.

23. Gebreheat G, Tsegay T, Kiros D, Teame H, Etsay N, Welu G, et al. Prevalence and Associated Factors of Perinatal Asphyxia among Neonates in General Hospitals of Tigray, Ethiopia, 2018. BioMed research international. 2018;2018:7.

24. Alemu A, Melaku G, Abera GB, Damte A. Prevalence and associated factors of perinatal asphyxia among newborns in Dilla University referral hospital, Southern Ethiopia- 2017. Pediatric Health Med Ther. 2019;10:69-74.

25. FDRE Ministry of Health. National Reproductive Health Strategy. Addis Ababa, Ethiopia: Ministry of Health; 2016. p. 100.

26. <Silte Zone Health department.pdf>.

27. Federal Ministry of Health. Neonatal Intensive Care Unit (NICU) Training. Management Protocol. Addis Ababa2014. p. 137.

28. Ibrahim NA, Muhye A, Abdulie S. Prevalence of Birth Asphyxia and Associated Factors among Neonates Delivered in Dilchora Referral Hospital, in Dire Dawa, Eastern Ethiopia. Clinics in Mother and Child Health. 2017;14(4).

29. Selvakumar R VC, Deepthy SIJ Incidence, Severity and Early Outcome of Hypoxicischemic Encephalopathy among Newborns Born in a Rural Tertiary Care Centre in Southern India. International Journal of Scientific Study. 2017;5(8):63-66.

30. Berglund S, Grunewald C, Pettersson H, Cnattingius S. Risk factors for asphyxia associated with substandard care during labor. Acta Obstet Gynecol Scand. 2010;89(1):39-48.

31. Wood S, Crawford S, Hicks M, Mohammad K. Hospital-related, maternal, and fetal risk factors for neonatal asphyxia and moderate or severe hypoxic-ischemic encephalopathy: a retrospective cohort study. J Matern Fetal Neonatal Med. 2019:1-6.

32. Ekwochi U, Asinobi NI, Osuorah CD, Ndu IK, Ifediora C, Amadi OF, et al. Incidence and Predictors of Mortality Among Newborns With Perinatal Asphyxia: A 4-Year Prospective Study of Newborns Delivered in Health Care Facilities in Enugu, South-East Nigeria. Clin Med Insights Pediatr. 2017;11:1-10.

33. Solayman M HS, Akber T , Islam MI , Islam MA. Prevalence of Perinatal Asphyxia with Evaluation of Associated Risk Factors in a Rural Tertiary Level Hospital. KYAMC Journal. 2017;8(1):43-48.

34. Samba A. Birth Asphyxia among Term Neonates at Korle-Bu Teaching Hospital (KBTH) in Accra. Obstetrics & Gynecology International Journal. 2017;7(6):1-4.

35. Gudayu TW. Proportion and factors associated with low fifth minute Apgar score among singleton newborn babies in Gondar University referral hospital; North West Ethiopia. African health sciences. 2017;17(1):1-6.

36. Abdo RA, Halil HM, Kebede BA, Anshebo AA, Gejo NG. Prevalence and contributing factors of birth asphyxia among the neonates delivered at Nigist Eleni Mohammed memorial teaching hospital, Southern Ethiopia: a cross-sectional study. BMC pregnancy and childbirth. 2019;19(1):536.

37. Nguanboonmak A SS. Prevalence and Risk Factors of Birth Asphyxia among Elderly Gravidarum. Thai Jour of Obst and Gynae. 2019;27(1):29-37.

38. Ilah B, Aminu M, Musa A, Adelakun M, Adeniji A, Kolawole T. Prevalence and Risk Factors for Perinatal Asphyxia as Seen at a Specialist Hospital in Gusau, Nigeria. Sub-Saharan African Journal of Medicine. 2015;2(2):64-68.

39. Royal College of Obstetricians and Gynaecologists. Antepartum Haemorrhage Green–top Guideline. UK: RCOG; 2011. p. 1-15.

40. Maternal and perinatal outcome of antepartum hemorrhage at three teaching hospitals in Addis Ababa, Ethiopia. Ethiopian Journal of Reproductive Health. 2020;12(3):1-19.

41. Hagos AA, Teka EC, Degu G. Utilization of Partograph and its associated factors among midwives working in public health institutions, Addis Ababa City Administration,Ethiopia,2017. BMC pregnancy and childbirth. 2020;20(1):49.

42. Worku AG, Yalew AW, Afework MF. Maternal complications and women's behavior in seeking care from skilled providers in North Gondar, Ethiopia. PloS one. 2013;8(3):e60171.

43. Utomo MT E, Winardi B Maternal Risk Factors Of Premature Delivery In Dr Soetomo Hospital. Folia Medica Indonesiana. 2011;47(1):1-45.

44. Woticha EW, Deressa W, Reja A. Barriers for detection and management of gestational diabetes mellitus in southern Ethiopia: A qualitative study. Diabetes Metab Syndr. 2019;13(3):1827-1831.

45. Muche AA, Olayemi OO, Gete YK. Gestational diabetes mellitus increased the risk of adverse neonatal outcomes: A prospective cohort study in Northwest Ethiopia. Midwifery. 2020;87:1-9.

46. Kiyani AN KA, Ehsan A Perinatal Factors Leading to Birth Asphyxia among Term Newborns in a Tertiary Care Hospital. Iran J Pediatr. 2014;24(5):637-642.

47. Mitao M, Philemon R, Obure J, Mmbaga BT, Msuya S, Mahande MJ. Risk factors and adverse perinatal outcome associated with low birth weight in Northern Tanzania: a registry-based retrospective cohort study. Asian Pacific Journal of Reproduction. 2016;5(1):75-79.

48. Borna S, Borna H, Khazardoost S, Hantoushzadeh S. 'Perinatal outcome in preterm premature rupture of membranes with Amniotic fluid index < 5 (AFI < 5). BMC pregnancy and childbirth. 2004;4(1):15.

49. Tewesa E, Chirwa E, Majamanda MD, Maluwa A, Chimwaza A. Associative Factors for Birth Asphyxia at Queen Elizabeth Central Hospital—Malawi. Journal of Biosciences and Medicines. 2017;05(05):22-31.

50. Mehta V, E A, Spoorthi, Archana, Hassan M. Meconium-stained amniotic fluid as a risk factor for perinatal asphyxia. International Journal of Contemporary Pediatrics. 2019;6(2).

51. Gebregziabher GT, Hadgu FB, Abebe HT. Prevalence and Associated Factors of Perinatal Asphyxia in Neonates Admitted to Ayder Comprehensive Specialized Hospital, Northern Ethiopia: A Cross-Sectional Study. Int J Pediatr. 2020;2020:1-8.

52. Engida Yisma1 BD, Ayalew Astatkie, Nebreed Fesseha. Knowledge and utilization of partograph among obstetric care givers in public health institutions of Addis Ababa, Ethiopia. BMC Pregnancy & Childbirth. 2013;13(17):1-9.

53. Haile Y, Tafese F, Weldemarium TD, Rad MH. Partograph Utilization and Associated Factors among Obstetric Care Providers at Public Health Facilities in Hadiya Zone, Southern Ethiopia. J Pregnancy. 2020;2020:8.

54. Markos M, Arba A, Paulos K. Partograph Utilization and Associated Factors among Obstetric Care Providers Working in Public Health Facilities of Wolaita Zone, 2017. J Pregnancy. 2020;2020:8.

55. Wakgari N, Amano A, Berta M, Tessema GA. Partograph utilization and associated factors among obstetric care providers in North Shoa Zone, Central Ethiopia: a cross sectional study. African health sciences. 2015;15(2):552-559.

Date November 5, 2021

Title: - Perinatal asphyxia and associated factors among neonates admitted to a specialized public hospital in South Central Ethiopia: A retrospective cross-sectional study

Manuscript reference number: PONE-D-21-06937R1

From: Authors

To: Academic Editor

Version: II

Subject: Revision of the manuscript

We profoundly acknowledge the efforts and resources put to review our work. We have accepted all the comments and constructive feedbacks given by the reviewers. We revised the paper comprehensively and responded in detail to the questions and comments from the reviewers. We have highlighted the changed areas using the Microsoft word 'Track Changes' option. The point-by-point description of the changes is provided below.

Best regards,

Authors

Responses for the Academic editor

Comment 1: For the reader an explanation of the 3 delays model would be helpful.

Response 1: We respectfully accepted the comment. It was briefly explained in the Discussion.

Comment 2: Please give more detailed information regarding possible different infections of the fetus and the neonate.

Response 2: We respectfully accepted the comment. We have noted that the statement put in the Discussion was ambigious. However the statemet was put as a rationale for the similarity between ours and other studies. We also have tried to correct it as such on the revised document.

Comment 3: What umbilical cord compromises have occured in this patient sample?

Response 3: We thank you for the question. We have noted that the statement put in the Discussion was ambigious. However the statemet was placed as a rationale for the similarity between ours and other studies. We also have tried to correct it as such on the revised document.

Comment 4: What percent of reduction of neonatal mortality do you aim for, what is feasible?

Response 4: We thank you for the question. In alignment with the sustainable development goals, we aim for a 35-40% reduction in the magnitude of perinatal asphyxia at WCHS by the end of the decade.

Comment 5: Please explain the capabilities of the different nurseries, especially the NICU - levels of care 1-4.

Response 5: We respectfully accepted the comment. It was briefly discussed in the Methods section, 'Study area' sub-section of the revised document.

Comment 6: Most importantly: Please review your paper, most of all the discussion, for unnecessary redundancies, length of text.

Response 6: We respectfully accepted the comment. We have tried to omit redundancies throughout the discussion section.

Thank you again!

Responses for Reviewer 1

Comment 1: Much improved manuscript.

Response 1: We are thankful once again for taking your time to refine our work.

Comment 2: Line 26 - delete "in the world" as this is repetitive.

Response 2: We respectfully accepted the comment. We have omitted the phrase.

Comment 3: Line 334 - change to read "Utilization of the APGAR alone to diagnoses perinatal asphyxia may not..."

Response 3: We respectfully accepted the comment. We have corrected it as per your recommendation.

Thank you again!

Responses for Reviewer 2

Comment 1: The paper has been significantly improved after revising. It covers an important topic and is well written.

Response 1: We thank you for the encouraging words and the time you have spent in refining the document.

Comment 2: In Abstract (page 2 lines 29-30), the authors stated that " ...studies on the prevalence of asphyxia in these countries are limited." The above statement is incorrect. This is not new information. Many other similar studies have been conducted in Ethiopia and published. It is unclear how this study specifically contributes to the literature. Suggest double-checking the similarity report too.

Response 2: We respectfully accepted the comment. As per your recommendation, a correction was made to the specific statement that indicated there is limited information on the topic. We also believe the study's findings will add to the body of knowledge interms of illuminating the problem in the study area. Furthermore, the findings of this study will direct responsible federal and local stakeholders to design effective implementations to reduce the magnitude of perinatal asphyxia.

Comment 3: Suggest consolidating the Introduction. The operational definition should be elaborated further in this section (not Methodology).

Response 3: We respectfully accepted the comment. We added a paragraph on the diagnostic definitions of perinatal asphyxia in the Introduction. Further elaboration was added in the methods section, as there was a comment by other reviewer.

Comment 4: Methodology-A citation is needed for the study area and period (Page 4, line 97)

Response 4: We respectfully accepted the comment. A citation is added to the description of the study area.

Comment 5: Please revise the inclusion criteria as it is similar to the source population. (page 5, line 105 & line 110). Please take note that "2016 to December 31, 2019" appeared three times in Methods.

Response 5: We respectfully accepted the comment. We have tried to reduce the redundancies by compiling only the necessary information under the sub section 'Study population'.

Comment 6: Please remove this statement "The overall prevalence of perinatal asphyxia among neonates who were delivered at WCHS was found to be 128(41.2%) from Clinical Characteristics" (page11, lines 204-205). You have reported it under the magnitude of perinatal asphyxia (Page 12, lines 228-229).

Response 6: We respectfully accepted the comment. As per your recommendation, the statement is removed in the revised editon.

Comment 7: The Discussion has improved. Suggest to re-write Limitation because it does not appear to be sound.

Response 7: We respectfully accepted the comment. We have tried to modify the Limitation in the revised edition.

Comment 8: The conclusion can be further enhanced by adding recommendations for future research.

Response 8: We respectfully accepted the comment. We added recommendations for future researchers in the revised document.

Thank you again!
