# Supplementary material for: Perinatal asphyxia and associated factors among neonates admitted to a specialized public hospital in South Central Ethiopia: A retrospective cross-sectional study
Source: PLoS One. 2022 Jan 13;17(1):e0262619. doi: 10.1371/journal.pone.0262619 (PMC8758104; doi:10.1371/journal.pone.0262619)
Supplement: Attachment — Submitted filename: Plos One_ Perinatal Asphyxia_Cpl.docx [file pone.0262619.s001.docx]

**Perinatal asphyxia and associated factors among neonates admitted to a specialized public hospital in South Central Ethiopia: A retrospective cross-sectional study.**

Thank you for the opportunity to review this manuscript.

Below are comments/suggestions for the authors' consideration.

The article is well written, highlights an actual problem ( perinatal asphyxia ), and uncovers factors associated with perinatal asphyxia.

The abstract is concise and accurately summarizes the essential information of the paper.

The introduction is appropriate for the content of the article although it would be better if the authors include a paragraph to detail the

(i) background introduction information of Ethiopia relevant to this topic.
(ii) associated risk factors to perinatal asphyxia

The methodology can be further enhanced:

-- The study was conducted at Worabe Comprehensive Specialized Hospital (WCSH), south-central Ethiopia, perhaps the reader would appreciate it if the authors could detail the study setting.

-- The authors have mentioned the systematic random sampling method. (page 5, line 106), this should be elaborated further on the systematic random sampling method used in Data Collection Procedure. ( Page 5, line 115-119)

--Description of the checklist.

--May need to include the IRB ethical approval number in the text.

--Number of records that meet the inclusion criteria. & the number and reasons of records excluded in this study.

Data analysis procedures are sufficiently described. Results are organized in a way that is easy to understand. The statistics are reported appropriately. Corrections need for :

The total number of occupational statuses is more than 311. (Table 1)

Please add notes explaining any acronyms or abbreviations in the table. (Table 2 & Table 4)

Revise no illness as it is misleading ( Table 2)

The author has reported the frequency of mothers of asphyxiated neonates is 128 ( Table 1). This is incongruent with Table 3 Clinical characteristics of neonates with perinatal asphyxia, the researcher has reported 119 in the yes category. Please recheck.

In reporting of factor associated with perinatal asphyxia, it would be more meaningful to report

e.g ...neonates from mothers who had antepartum hemorrhage were 4.5 times more affected by asphyxia as compared to neonates who delivered from mothers who had no antepartum hemorrhage(AOR= 4.5, 95%CI, 2.3,8.6) (Page 10 . line 188-189)

Table 4: Please add notes * p<0.05

The discussion and conclusion are well articulated. However, it was rather scanty. Suggest to elaborate further on “ This major difference could be attributed to the methodological approaches employed amongst the studies, the definition of birth asphyxia, and the management protocol of the hospitals for perinatal asphyxia. (Page 13, line 225-226) & as appropriate. And the study limitations are not discussed.

However, the references/ citations are appropriate.

Thank you
