# Supplementary material for: Perinatal asphyxia and associated factors among neonates admitted to a specialized public hospital in South Central Ethiopia: A retrospective cross-sectional study
Source: PLoS One. 2022 Jan 13;17(1):e0262619. doi: 10.1371/journal.pone.0262619 (PMC8758104; doi:10.1371/journal.pone.0262619)
Supplement: Attachment — Submitted filename: Response to Reviewers.docx [file pone.0262619.s002.docx]

**Date August 31, 2021**

**Title: - Perinatal asphyxia and associated factors among neonates admitted to a specialized public hospital in South Central Ethiopia: *A retrospective cross sectional study***

Manuscript reference number: PONE-D-21-06937

From: Authors

To: Academic Editor

Version: I

Subject: Revision of the manuscript

We are very thankful for the comments and constructive feedbacks given by the reviewers. We profoundly acknowledge the efforts and resources put to review our work. We revised the paper comprehensively and responded in detail to the questions and comments from the reviewers. We have highlighted the changed areas using the Microsoft word ‘Track Changes’ option. The point-by-point description of the changes is provided below.

Best regards,

Authors

**Responses for the Academic editor**

**Comment 1**: Please ensure that your manuscript meets PLOS ONE's style requirements, including those for file naming.

**Response 1:** We respectfully accepted the comment. The submitted version is corrected according to the PLOS ONE's style requirements.

**Comment 2**: Please ensure you have discussed any potential limitations of your study in the Discussion, including study design, sample size and/or potential confounders.

**Response 2:** We respectfully accepted the comment. The revised version added the limitations of this study after the discussion section.

**Comment 3**: Please provide additional details regarding participant consent. In the ethics statement in the Methods and online submission information, please ensure that you have specified (1) whether consent was informed and (2) what type you obtained (for instance, written or verbal, and if verbal, how it was documented and witnessed). If your study included minors, state whether you obtained consent from parents or guardians. If the need for consent was waived by the ethics committee, please include this information.

If you are reporting a retrospective study of medical records or archived samples, please ensure that you have discussed whether all data were fully anonymized before you accessed them and/or whether the IRB or ethics committee waived the requirement for informed consent. If patients provided informed written consent to have data from their medical records used in research, please include this information.

**Response 3:** We respectfully accepted the comment. The anonymity of the study participants was kept confidential throughout the entire process. We have also added a statement pertaining to the comments under the section ‘Ethical considerations’.

**Responses for Reviewer 1**

**First of all, we are very grateful for your encouraging words. We are humbled by the constructive feedback and the details you went through to review our work.**

***Responses for Reviewer 1 (Abstract)***

**Comment 1**: Line 29 – Edit to “Perinatal asphyxia continues to be a significant clinical concern around the world as the consequences can be devastating.”

**Response 1:** We respectfully accepted the comment. It was edited on the revised document.

**Comment 2**: Include that the charts were reviewed from a 3 year period.

**Response 2:** We respectfully accepted the comment. It was edited on the revised document.

***Responses for Reviewer 1 (Introduction)***

**Comment 3**: Line 55 delete the word “only”

**Response 3:** We respectfully accepted the comment. It was deleted on the revised document.

**Comment 4**: Rework 55-60. Try this: 56-60 Perinatal asphyxia, also called birth asphyxia, is one of the leading causes of neonatal deaths in the world following severe infections and prematurity (1, 4). It results from the loss of the blood supply or impairment of gas exchange to or from the fetus before, during, or after the birth process (5). Perinatal asphyxia may lead to severe metabolic acidosis, hypercarbia, progressive hypoxemia, neonatal encephalopathy, and multi-system organ failure, and even result in death (6-10).

**Response 4:** We respectfully accepted the comment. It was edited as such on the revised document.

**Comment 5**: Line 70 delete the word “staggering”

**Response 5:** We respectfully accepted the comment. It was deleted from the revised document.

**Comment 6**: Line 71 Is this the name of a program? If so, it should all be capitalized. National Strategy for Newborn and Child Survival

**Response 6:** We respectfully accepted the comment. Yes, it is a name of a program and a correction was made.

**Comment 7**: Rework 77-82. Try this: 77-82 In order to improve the quality of care that is delivered within a country, one must first understand the problem and the issues that healthcare providers encounter. Improving care of the maternal-infant dyad can lead to a significant decrease in the rates of perinatal asphyxia (find a reference for this). In an effort to improve care in Ethiopia, we sought to understand the burden of perinatal asphyxia and early assessment practices. To do this, we evaluated cases of perinatal asphyxia and identified associated factors among neonates admitted to the neonatal intensive care unit of Worabe Comprehensive Specialized Hospital in southern Ethiopia

**Response 7:** We respectfully accepted the comment. It was edited as such on the revised document.

***Responses for Reviewer 1 on Methods***

**Comment 8**: This whole section is a little unclear. Were all records reviewed? This is what it sounds like from the first few lines. Or, were only 311 records reviewed based on some sampling procedure? This is what it sounds like as you read on.

**Response 8:** We respectfully accepted the comment and question. The records that were reviewed were 311 out of the total neonatal records (3796) from the three years period. We used systematic random sampling technique to select the records. This was incorporated in the manuscript to make it clear.

**Comment 9**: The section: “Sample size determination and sampling procedure” needs to be rewritten.

**Response 9:** We respectfully accepted the comment. The sub-section is rewritten with more details.

**Comment 10**: I need more information about what is being seen at Worabe in order to know if this even makes sense. How many births occurred there during the three-year period? How many are admitted to the NICU over the three-year period? How did you randomly select which charts to review? It is all very vague.

**Response 10:** We respectfully accepted the comment and questions. The total birth at Worabe hospital during the three years period was 10,800 and 3796 newborns were admitted to the NICU within this time frame. Systematic random sampling technique was used to select which charts to review. After the card records of the neonates were put in their order of admission, the k^th^ interval was determined by dividing the total population size by the total sample size.

**Comment 11**: The biggest thing that is lacking is how was perinatal asphyxia defined? I am worried there was no uniform definition and a Sarnat 1 baby is being included in with Sarnat 2 and 3 babies. While technically all three are encephalopathies (stages of it), outcomes are very different and your information would be strengthened if we knew the definition and the range of severity in your definition.

**Response 11:** We respectfully accepted the comment and questions. In our study, perinatal asphyxia was defined as the inability of a newborn to initiate and sustain respiration, by scoring an APGAR score less than 7 persistently for more than 5 min after delivery.

***Responses for Reviewer 1 on Results***

**Comment 12**: Line 167 “developed respiratory distress during labor.” This seems to be referencing the baby. How does a baby develop respiratory distress during labor? Looking at Table 3, I believe you mean the baby developed perinatal asphyxia during labor.

**Response 12:** We respectfully accepted the comment and questions. It was an error during the preparation of the manuscript. The phrase on line 167 “developed respiratory distress during labor” was to mean “fetal distress”. What was presented in Table 3 also is corrected as “fetal distress” instead of perinatal asphyxia.

**Comment 13**: Line 168 – where does it say this in the table?

**Response 13:** We respectfully accepted the comment. The percentage of neonates with and without perinatal asphyxia was incorporated into Table 3. The magnitude of perinatal asphyxia was indeed 41.2%. These errors are corrected on the revised manuscript.

**Comment 14**: Line 172,173 – the table says it is 119 and 38.3%

**Response 14:** We respectfully accepted the comment. It was an error and corrected as per the edition made to Table 3.

***Responses for Reviewer 1 on Discussion***

**Comment 15**: Line 225 – “the definition of birth asphyxia.” This is key. What is the definition for this study? I didn’t see it defined.

**Response 15:** We respectfully accepted the comment. The operational definitions for this study are added to the revised document.

**Comment 16**: Overall, I think the discussion could use significant work rather than just stating that you found what others did. I would expect that to happen as these are all well-documented factors associated with perinatal asphyxia.

**Response 16:** We respectfully accepted the comment. We added evidence-based supplements to the discussion section to modify it.

**Comment 17**: What I would really like to see done in this section is how could this information be applied in Ethiopia to improve care? How could you use this information to design an educational intervention and then measure the impact? Don’t just draw conclusions from the data, but let it lead you somewhere.

**Response 17:** We respectfully accepted the comment. The section was modified to address the concerns raised and it was contextualized based on the comments given.

**Comment 18**: I also need you to think about what the limitations of your study might be. One I see are the potential problems with your data. Was it good? What about how the clinicians defined perinatal asphyxia? I am worried that your numbers are so high because of a variation in diagnosis amongst the providers.

**Response 18:** We respectfully accepted the comment. The revised version incorporated the limitations of this study after the discussion section.

***Responses for Reviewer 1 on Conclusion***

**Comment 19**: Based on the above feedback, the conclusion would also need to be reworked.

**Response 19:** Thank you for the comment. The conclusion is rewritten in accordance with the discussion.

**Thank you again!**

**Responses for Reviewer 2**

**First of all, we are very grateful for your encouraging words. We are humbled by the constructive feedback and the details you went through to review our work.**

***Responses for Reviewer 2 on Introduction***

**Comment 1**: The introduction is appropriate for the content of the article although it would be better if the authors include a paragraph to detail the

(i) background introduction information of Ethiopia relevant to this topic.

(ii) associated risk factors to perinatal asphyxia

**Response 1:** We respectfully accepted the comment. These components are incorporated in the revised manuscript.

***Responses for Reviewer 2 on Methods***

**Comment 2**: The study was conducted at Worabe Comprehensive Specialized Hospital (WCSH), south-central Ethiopia, perhaps the reader would appreciate it if the authors could detail the study setting.

**Response 2:** We respectfully accepted the comment. We have included additional data pertaining to the study setting.

**Comment 3**: The authors have mentioned the systematic random sampling method. (page 5, line 106), this should be elaborated further on the systematic random sampling method used in Data Collection Procedure. ( Page 5, line 115-119)

**Response 3:** We respectfully accepted the comment. We have segregated the sub-section “Sample size determination and sampling procedure” into two. We explained the details on how we used the systematic random sampling under the “Sampling procedure” sub-section.

**Comment 4**: Description of the checklist.

**Response 4:** We respectfully accepted the comment. We have tried to describe the checklists used in this study under Data collection tools and procedures.

**Comment 5**: May need to include the IRB ethical approval number in the text.

**Response 5:** We respectfully accepted the comment. We have incorporated the ethics approval number granted by School of Nursing and Midwifery Research Ethical Committee into the revised document.

**Comment 6**: Number of records that meet the inclusion criteria. & the number and reasons of records excluded in this study.

**Response 6:** We respectfully accepted the comment. The calculated sample size was 311 and all randomly selected records fulfilled the inclusion criteria. We have not excluded a randomly selected record because we have robustly searched for other sources of data (to trace and find any missing information) including card room registration book, referral paper, ward admission and discharge registration book and documented nursing care plans.

***Responses for Reviewer 2 on Results***

**Comment 7**: The total number of occupational statuses is more than 311. (Table 1)

**Response 7:** We respectfully accepted the comment. A correction is made to the revised document.

**Comment 8**: Please add notes explaining any acronyms or abbreviations in the table. (Table 2 & Table 4)

**Response 8:** We respectfully accepted the comment. Notes explaining the acronyms or abbreviations are put under Tables 2 and 4.

**Comment 9**: Revise no illness as it is misleading (Table 2)

**Response 9:** We respectfully accepted the comment. It is corrected as chronic illness on the revised manuscript.

**Comment 10**: The author has reported the frequency of mothers of asphyxiated neonates is 128 ( Table 1). This is incongruent with Table 3 Clinical characteristics of neonates with perinatal asphyxia, the researcher has reported 119 in the yes category. Please recheck.

**Response 10:** We respectfully accepted the comment. The percentage of neonates with and without perinatal asphyxia was incorporated into Table 3. The magnitude of perinatal asphyxia was indeed 128 (41.2%). These errors are corrected in the revised manuscript.

**Comment 10**: In reporting of factor associated with perinatal asphyxia, it would be more meaningful to report e.g ...neonates from mothers who had antepartum hemorrhage were 4.5 times more affected by asphyxia as compared to neonates who delivered from mothers who had no antepartum hemorrhage(AOR= 4.5, 95%CI, 2.3,8.6) (Page 10 . line 188-189)

**Response 10:** We respectfully accepted the comment. It is corrected on the revised manuscript.

**Comment 11**: Table 4: Please add in notes * p<0.05

**Response 11:** We respectfully accepted the comment. The value of significance is added to the revised manuscript.

**Comment 12**: The discussion and conclusion are well articulated. However, it was rather scanty.

**Response 12:** We respectfully accepted the comment. Based on the suggestion, more content is added to enrich the discussion.

**Comment 13**: Suggest to elaborate further on “This major difference could be attributed to the methodological approaches employed amongst the studies, the definition of birth asphyxia, and the management protocol of the hospitals for perinatal asphyxia (Page 13, line 225-226) & as appropriate.

**Response 13:** We respectfully accepted the comment. These points are plausibly elaborated in the revised document.

**Comment 14**: And the study limitations are not discussed. However, the references/ citations are appropriate.

**Response 14:** We respectfully accepted the comment. The revised version incorporated the limitations of this study after the discussion section.

**Thank you again!**
