# Supplementary material for: Perinatal asphyxia and associated factors among neonates admitted to a specialized public hospital in South Central Ethiopia: A retrospective cross-sectional study
Source: PLoS One. 2022 Jan 13;17(1):e0262619. doi: 10.1371/journal.pone.0262619 (PMC8758104; doi:10.1371/journal.pone.0262619)
Supplement: Attachment — Submitted filename: Plos One_ Perinatal Asphyxia_R1.docx [file pone.0262619.s003.docx]

**Perinatal asphyxia and associated factors among neonates admitted to a specialized public hospital in South Central Ethiopia: A retrospective cross-sectional study.**

The paper has been significantly improved after revising.

It covers an important topic and is well written. In Abstract (page 2 lines 29-30), the authors stated that “ ...studies on the prevalence of asphyxia in these countries are limited.” The above statement is incorrect. This is not new information. Many other similar studies have been conducted in Ethiopia and published. It is unclear how this study specifically contributes to the literature. Suggest double-checking the similarity report too.

- <https://journals.plos.org/plosone/article?id=10.1371/journal.pone.0255488> Prevalence and risk factors associated with birth asphyxia among neonates delivered in Ethiopia: A systematic review and meta-analysis

# doi: [10.1016/j.heliyon.2020.e03793](https://dx.doi.org/10.1016%2Fj.heliyon.2020.e03793) Prevalence of perinatal asphyxia in East and Central Africa: systematic review and meta-analysis

- DOI: [10.1155/2020/4367248](http://dx.doi.org/10.1155/2020/4367248) Prevalence and Associated Factors of Perinatal Asphyxia in Neonates Admitted to Ayder Comprehensive Specialized Hospital, Northern Ethiopia: A Cross-Sectional Study

# doi: [10.1371/journal.pone.0226891](https://dx.doi.org/10.1371%2Fjournal.pone.0226891) Birth asphyxia and its associated factors among newborns in public hospital, northeast Amhara, Ethiopia

# <https://bmcpediatr.biomedcentral.com/articles/10.1186/s12887-021-02598-z> Neonatal mortality among neonates admitted to NICU of Hiwot Fana specialized university hospital, eastern Ethiopia, 2020: a cross-sectional study design

- DOI: [10.1155/2018/5351010](https://doi.org/10.1155/2018/5351010) Prevalence and Associated Factors of Perinatal Asphyxia among Neonates in General Hospitals of Tigray, Ethiopia, 2018

# doi: [10.2147/PHMT.S196265](https://dx.doi.org/10.2147%2FPHMT.S196265) Prevalence and associated factors of perinatal asphyxia among newborns in Dilla University referral hospital, Southern Ethiopia– 2017

# Suggest consolidating the Introduction. The operational definition should be elaborated further in this section (not Methodology).

# Methodology-A citation is needed for the study area and period (Page 4, line 97)

# Please revise the inclusion criteria as it is similar to the source population. (page 5, line 105 & line 110). Please take note that “2016 to December 31, 2019” appeared three times in Methods.

# Please remove this statement “The overall prevalence of perinatal asphyxia among neonates who were delivered at WCHS was found to be 128(41.2%) from Clinical Characteristics” (page11, lines 204-205). You have reported it under the magnitude of perinatal asphyxia (Page 12, lines 228-229).

The Discussion has improved. Suggest to re-write Limitation because it does not appear to be sound. The conclusion can be further enhanced by adding recommendations for future research.
