# Supplementary material for: Perinatal asphyxia and associated factors among neonates admitted to a specialized public hospital in South Central Ethiopia: A retrospective cross-sectional study
Source: PLoS One. 2022 Jan 13;17(1):e0262619. doi: 10.1371/journal.pone.0262619 (PMC8758104; doi:10.1371/journal.pone.0262619)
Supplement: Attachment — Submitted filename: Response to Reviewers.docx [file pone.0262619.s004.docx]

**Date November 5, 2021**

**Title: - Perinatal asphyxia and associated factors among neonates admitted to a specialized public hospital in South Central Ethiopia: A retrospective cross-sectional study**

Manuscript reference number: PONE-D-21-06937R1

From: Authors

To: Academic Editor

Version: II

Subject: Revision of the manuscript

We profoundly acknowledge the efforts and resources put to review our work. We have accepted all the comments and constructive feedbacks given by the reviewers. We revised the paper comprehensively and responded in detail to the questions and comments from the reviewers. We have highlighted the changed areas using the Microsoft word ‘Track Changes’ option. The point-by-point description of the changes is provided below.

Best regards,

Authors

**Responses for the Academic editor**

**Comment 1**: For the reader an explanation of the 3 delays model would be helpful.

**Response 1:** We respectfully accepted the comment. It was briefly explained in the Discussion.

**Comment 2**: Please give more detailed information regarding possible different infections of the fetus and the neonate.

**Response 2:** We respectfully accepted the comment. We have noted that the statement put in the Discussion was ambigious. However the statemet was put as a rationale for the similarity between ours and other studies. We also have tried to correct it as such on the revised document.

**Comment 3**: What umbilical cord compromises have occured in this patient sample?

**Response 3:** We thank you for the question. We have noted that the statement put in the Discussion was ambigious. However the statemet was placed as a rationale for the similarity between ours and other studies. We also have tried to correct it as such on the revised document.

**Comment 4**: What percent of reduction of neonatal mortality do you aim for, what is feasible?

**Response 4:** We thank you for the question. In alignment with the sustainable development goals, we aim for a 35-40% reduction in the magnitude of perinatal asphyxia at WCHS by the end of the decade.

**Comment 5**: Please explain the capabilities of the different nurseries, especially the NICU - levels of care 1-4.

**Response 5:** We respectfully accepted the comment. It was briefly discussed in the Methods section, ‘Study area’ sub-section of the revised document.

**Comment 6:** Most importantly: Please review your paper, most of all the discussion, for unnecessary redundancies, length of text.

**Response 6:** We respectfully accepted the comment. We have tried to omit redundancies throughout the discussion section.

**Thank you again!**

**Responses for Reviewer 1**

**Comment 1:** Much improved manuscript.

**Response 1:** We are thankful once again for taking your time to refine our work.

**Comment 2**: Line 26 - delete "in the world" as this is repetitive.

**Response 2:** We respectfully accepted the comment. We have omitted the phrase.

**Comment 3**: Line 334 - change to read "Utilization of the APGAR alone to diagnoses perinatal asphyxia may not..."

**Response 3:** We respectfully accepted the comment. We have corrected it as per your recommendation.

**Thank you again!**

**Responses for Reviewer 2**

**Comment 1**: The paper has been significantly improved after revising. It covers an important topic and is well written.

**Response 1:** We thank you for the encouraging words and the time you have spent in refining the document.

**Comment 2**: In Abstract (page 2 lines 29-30), the authors stated that “ ...studies on the prevalence of asphyxia in these countries are limited.” The above statement is incorrect. This is not new information. Many other similar studies have been conducted in Ethiopia and published. It is unclear how this study specifically contributes to the literature. Suggest double-checking the similarity report too.

**Response 2:** We respectfully accepted the comment. As per your recommendation, a correction was made to the specific statement that indicated there is limited information on the topic. We also believe the study’s findings will add to the body of knowledge interms of illuminating the problem in the study area. Furthermore, the findings of this study will direct responsible federal and local stakeholders to design effective implementations to reduce the magnitude of perinatal asphyxia.

**Comment 3**: Suggest consolidating the Introduction. The operational definition should be elaborated further in this section (not Methodology).

**Response 3:** We respectfully accepted the comment. We added a paragraph on the diagnostic definitions of perinatal asphyxia in the Introduction. Further elaboration was added in the methods section, as there was a comment by other reviewer.

**Comment 4**: Methodology-A citation is needed for the study area and period (Page 4, line 97)

**Response 4:** We respectfully accepted the comment. A citation is added to the description of the study area.

**Comment 5**: Please revise the inclusion criteria as it is similar to the source population. (page 5, line 105 & line 110). Please take note that “2016 to December 31, 2019” appeared three times in Methods.

**Response 5:** We respectfully accepted the comment. We have tried to reduce the redundancies by compiling only the necessary information under the sub section ‘Study population’.

**Comment 6**: Please remove this statement “The overall prevalence of perinatal asphyxia among neonates who were delivered at WCHS was found to be 128(41.2%) from Clinical Characteristics” (page11, lines 204-205). You have reported it under the magnitude of perinatal asphyxia (Page 12, lines 228-229).

**Response 6:** We respectfully accepted the comment. As per your recommendation, the statement is removed in the revised editon.

**Comment 7**: The Discussion has improved. Suggest to re-write Limitation because it does not appear to be sound.

**Response 7:** We respectfully accepted the comment. We have tried to modify the Limitation in the revised edition.

**Comment 8**: The conclusion can be further enhanced by adding recommendations for future research.

**Response 8:** We respectfully accepted the comment. We added recommendations for future researchers in the revised document.

**Thank you again!**
